# Exciton Coupling and Charge Transfer Dynamics in Zn(II) Complexes of π‑Extended Dipyrrins

**DOI:** 10.1021/acs.jpcb.5c03312

**Published:** 2025-06-13

**Authors:** Dabin Kim, Luca Ravotto, April Bialas, Thomas Troxler, Zhongping Ou, Karl M. Kadish, Andrei V. Cheprakov, Frank C. Spano, Sergei A. Vinogradov, Jessica M. Anna

**Affiliations:** † Department of Chemistry, School of Arts and Sciences, 6572 University of Pennsylvania, Philadelphia, Pennsylvania 19104, United States; ‡ Department of Biochemistry and Biophysics, Perelman School of Medicine, University of Pennsylvania, Philadelphia, Pennsylvania 19104, United States; § Department of Chemistry, Temple University, Philadelphia, Pennsylvania 19122, United States; ∥ Department of Chemistry, 14743 University of Houston, Houston, Texas 77204, United States; ⊥ Department of Chemistry, 64935 Moscow State University, 119899 Moscow, Russia; # Department of Chemistry, University of Pittsburgh, Pittsburgh, Pennsylvania 15216, United States

## Abstract

Dipyrrins form a group of versatile chromophores, which find use as laser dyes as well as in light harvesting and bioimaging applications. The mode of the central ion coordination and the ensuing molecular geometry play a key role in the photophysics of dipyrrins, whereby some complexes are brightly fluorescent and some completely lack emissivity. However, the relationship between the structure and excitation dynamics in dipyrrins is still poorly understood. Here, we used a range of spectroscopic methods to investigate the photophysics of Zn­(II) complexes of *meso*-Ar-2,2′-di-*tert*-butoxycarbonyl-dibenzodipyrrins (BDP; Ar = 4-MeO_2_C–C_6_H_4_). In particular, two-dimensional electronic spectroscopy (2DES) was used to characterize the initial excited states in a homoleptic *bis*-dipyrrinate Zn­(BDP)_2_, in which two dipyrrin ligands are oriented in a nonorthogonal geometry. From the position of the peaks in the 2DES spectra and spectral modeling, the initial excited states of Zn­(BDP)_2_ were assigned to excitonic states. The low oscillator strength, associated with excitation to the lower excitonic state, is responsible in part for the weak emissivity of Zn­(BDP)_2_, contrasting the bright fluorescence of *mono*-dippyrinate Zn­(BDP)­X. Femtosecond (fs-), nanosecond (ns-) transient absorption (TA), and time-resolved fluorescence spectroscopies were used to monitor the solvent-dependent evolution of the excitonic states, which appear to evolve into an intermediate state possibly with charge transfer character. Taken together, our findings reveal a significant impact of both structural and environmental factors on the photophysics of dipyrrins and present the first example of the application of 2DES to investigate excitonic states in a system where the interacting chromophores are held together via coordination of an optically neutral metal ion. On a broader scale, we demonstrate that nonorthogonal *bis*-dipyrrin complexes constitute a versatile model for studying exciton coupling and associated energy and charge dynamics.

## Graphical abstract

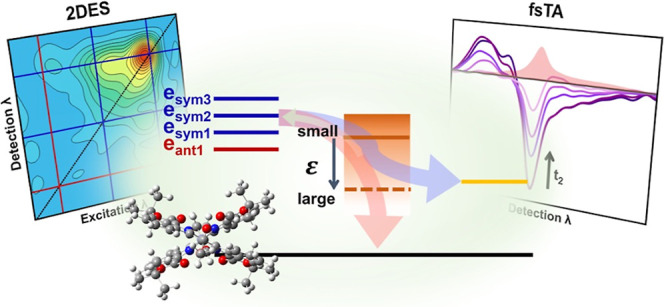

## Introduction

Dipyrromethenes, or dipyrrins, form a versatile class of organic dyes that present interest for a wide range of applications.^1–3^ For example, BODIPYs (boron difluoride dipyrromethenes)^4^ are broadly used as fluorescent sensors^5^ and have been proposed as photosensitizers for photodynamic therapy^6,7^ as well as electron donors/acceptors for artificial light-harvesting systems^8^ and organic photovoltaics.^9,10^ Dipyrrins can also serve as chelating ligands for metal ions.^1–3,11–14^ Previous studies have demonstrated that metallodipyrrin-based systems have the potential to operate as catalysts,^15^ photosensitizers,^16^ biosensors,^14,17–19^ and dyes for solar cells.^20,21^ The diversity of applications of dipyrrin complexes stems from their tunable electronic structure, whereby different photophysical pathways can be accessed through varying the substituents in the dipyrrin ligands, changing the metal ion, and/or altering the solvent environment.^2,14,22,23^ Studies that establish connections between the structure of dipyrrin complexes and their photophysical properties provide insight into how to manipulate and tune their photophysics for specific applications.

Dipyrrins can form *mono*- and *bis*-complexes with divalent metals.^1^ In *mono*-dipyrrinates, the second ligand is usually an optically inert anion, such as chloride, perchlorate or acetate. In *bis*-dipyrrinates, the ligands could be either two different or two identical dipyrrins, corresponding to heteroleptic and homoleptic complexes, respectively.^1,2^ Dipyrrin complexes with Zn^2+^, which is an optically inert closed-shell d_10_ ion, have been studied perhaps most widely. Bidentate ligation of a dipyrrin to Zn^2+^ acts to rigidify the dipyrrin skeleton, which is reflected by characteristic changes in the electronic absorption spectra. The photophysical properties of Zn *mono*-dipyrrinates resemble those of BODIPY,^4^ but the photophysics of Zn *bis*-dipyrrinates, where the Zn^2+^ ion serves essentially as a “glue” holding the two dipyrrin ligands together, is significantly different. In heteroleptic complexes, excitation energy can flow from a dipyrrin with a smaller π-system (higher excitation energy) to a larger one, and the latter can emit fluorescence.^24–26^ In homoleptic Zn *bis*-dipyrrinates, which, with rare exceptions,^11^ are almost nonemissive, the two dipyrrin units are typically orthogonal to one-another.^11,22,23,27,28^ However, in complexes of some dipyrrins, notably those possessing *meso*-aryl substituents and alkoxy-carbonyl groups in 2,2′-positions, the ligands’ planes are tilted relative to one-another by 50–70°.^29,30^

Fluorescence quenching in orthogonal homoleptic *bis*-dipyrrinates has been attributed to the existence of symmetry-breaking charge transfer (SBCT) states, which could mediate nonradiative relaxation to the ground and/or dark triplet states.^22,23,27,28,31–33^ In contrast, in nonorthogonal complexes, the lack of emissivity has been explained by excitonic coupling,^29,30^ based on the linear absorption spectra and fluorescence behavior, which were found to be consistent with the Kasha’s molecular exciton model.^33,34^ However, no studies of exciton dynamics in such systems have been reported.

The close proximity of two dipyrrin ligands with their transition dipole moments oriented in a nonorthogonal geometry could result in a nonzero Coulombic electronic coupling, leading to the formation of delocalized excitonic states.^29,34^ The low oscillator strength and, consequently, small radiative rate constant associated with the lower excitonic state alone could explain the drop in the emission quantum yield as well as the higher probability of intersystem crossing, which is further increased by the energetic proximity of the triplet and lower excitonic states.^34,35^ However, SBCT states could also play a role in the photophysics of nonorthogonal Zn dipyrrins by facilitating nonradiative decay and triplet formation.^22,32,33^

In this study, 2DES, fsTA, nsTA and fluorescence spectroscopies were used to characterize the excited state dynamics of *mono*- and *bis*-complexes of Zn with *meso*-Ar-2,2′-di-*tert*-butoxycarbonyl-dibenzodipyrrin (BDP; Ar = 4-MeO_2_C–C_6_H_4_) in order to determine the photophysical mechanisms that underpin the reduced quantum yield of fluorescence in Zn­(BDP)_2_, compared to monodipyrrinates, such as Zn­(BDP)­X (X = Cl, OAc, etc; Figure 1), as well as other spectroscopic differences between these complexes. From the positions of the cross-peaks in the 2DES spectra of Zn­(BDP)_2_ and spectral modeling with the Frenkel-Holstein Hamiltonian, the initial transitions in Zn­(BDP)_2_ were assigned as transitions to Frenkel excitonic states, confirming that exciton coupling plays a key role in the photophysics of nonorthogonal *bis*-dipyrrinates. The subsequent decay of the excitonic states was found to occur with the involvement of an intermediate transient state which could possess some charge transfer (CT) character, thus resembling SBCT states proposed for orthogonal Zn dipyrrins.^22^

**Figure 1:**
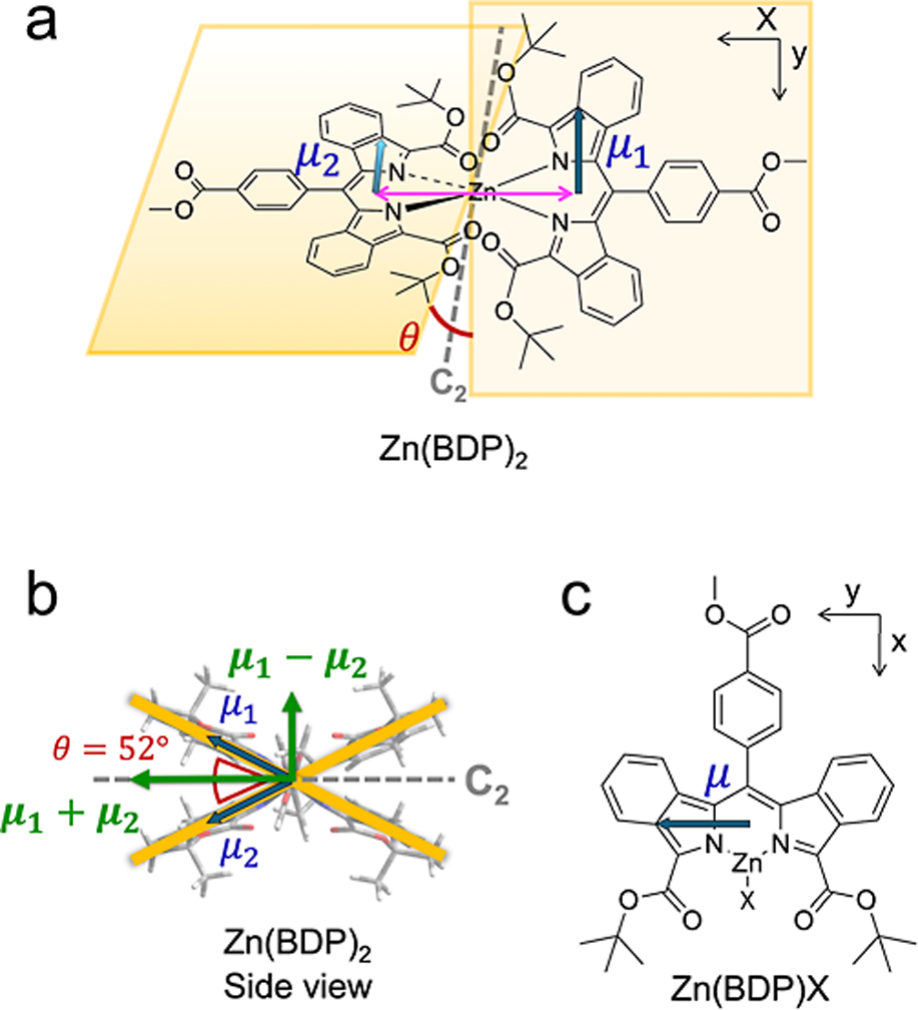
Computed molecular structures of Zn dibenzodipyrrin complexes. (a) Homoleptic Zn *bis*-dibenzodipyrrin, Zn­(BDP)_2_. The planes (highlighted in yellow) of the two BDP ligands are tilted relative to one-another at the dihedral angle θ = 52.66° (based on the computed structure: wB97XD/cc-pVTZ). The distance between the centroids of the two BDP units is 5.97 Å. The centroids were calculated for the dipyrrin skeletons with two fused benzo-rings (19 atoms), ignoring the substituents in the *meso*- and 2,2′-positions. The transition dipole moments of the two BDP ligands are indicated with blue arrows. (b) A sideview of Zn­(BDP)_2_ complex with the *C*_2_ symmetry axis (dashed brown line), which defines the symmetric (sym) and antisymmetric (ant) excitonic states and the corresponding transition dipole moments lying along the (**μ_1_** + **μ_2_**) and (**μ_1_** – **μ**_**2**_) directions. (c) Monodipyrrinate Zn­(BDP)­X (X = Cl·Pyr, OAc, etc.).

Significant attention has been attributed in recent years to the interplay between Frenkel excitons (FE), charge transfer excitons (CTE), and SBCT,^36,37^ reflecting the importance of these states and associated processes for the natural and artificial photosynthesis. However, the number of model systems suitable for experimental validation of the developing theoretical concepts remains limited. To the best of our knowledge, this work presents the first example of application of 2DES to chromophore “dimers” held together via complexation to an optically inert metal ion, where the 2DES provides critical information for demonstrating that b*is*-dipyrrinates make up a versatile, synthetically tunable, and structurally well-defined model for studies of excitonic coupling and associated energy and charge transfer processes.

## Methods

### Materials

All solvents were of the spectroscopic grade. Zn­(BDP)_2_ was prepared as described previously.^29^ Spectroscopic measurements were performed using solutions of Zn­(BDP)_2_ in four solvents of different polarity: acetonitrile (MeCN), diethyl ether (Et_2_O), toluene (TolH), and cyclohexane (cHex). Solutions of Zn­(BDP)Cl in dimethylformamide (DMF) or pyridine (Pyr) were obtained by adding ZnCl_2_ to a solution of free-base BDP in the corresponding solvent.^29^ Alternatively, a solution of Zn­(BDP)Cl was obtained by treating a solution of Zn­(BDP)_2_ in pyridine with excess of ZnCl_2_. In pyridine, the Zn^2+^ ion in monodipyrrinate is likely to be additionally coordinated by a pyridine molecule, forming a distorted tetrahedral complex (Supporting Information 3), Zn­(BDP)­Cl·Pyr. In the text below we omit Pyr in the abbreviations, referring to Zn^2+^ monodipyrrinate simply as Zn­(BDP)­Cl. Prior to measurements, solutions of Zn­(BDP)Cl were passed through syringe filters (Fisher, 0.2 μm, PTFE). Ideally, one would perform measurements on Zn­(BDP)Cl and Zn­(BDP)_2_ in the same solvent; however, this was not feasible, as Zn­(BDP)Cl and Zn­(BDP)_2_ have different stabilities/solubilities in different solvents.^29^

### Linear Absorption and Fluorescence Spectroscopy

UV/Vis spectra were recorded using Lambda 365 (PerkinElmer) and V-750 (Jasco) spectrophotometers. Fluorescence emission and excitation spectra were measured using a FS900 fluorometer (Edinburgh Instruments), as described previously.^29^ Emission spectra were obtained using solutions with absorbances at the excitation wavelengths of 0.02–0.05 OD. In all cases, the origin of the emitting species was confirmed by recording excitation spectra.

Fluorescence anisotropy measurements were performed using Zn­(BDP)_2_ dissolved in solid polymer films to ensure that the molecules did not undergo molecular reorientation over the course of the measurements. Silicone elastomer base (2.5 mL, Dow Chemicals) and catalyst (250 μL, Dow Chemicals) were mixed in a vial. A solution of Zn­(BDP)_2_ in toluene (750 μL, ∼1.5 × 10^–4^ M) was added to the mixture and slowly mixed in to avoid the formation of air bubbles. The resulting mixture was poured into a small aluminum tray (2 cm × 2 cm), which was covered with aluminum foil and placed on a heater plate for the film to cure at 80–100 °C for 2–3 h.

The film was mounted on a special holder designed to keep the sample at an angle relative to the beam, thus preventing reflection of the excitation light directly toward the detector. Excitation and emission polarizers were placed immediately before and after the film to minimize the loss of polarization. Two long-pass filters, 780 nm (XIL0780, Asahi Spectra and SCHOTT RG780, Edmund Optics) were mounted in front of the slits of the emission monochromator. The slits were additionally guarded by a custom-made narrow light channel that permitted access of the ballistic photons coming directly from the sample, while preventing access of the scattered light. This measure was important in view of the very low emission quantum yield of Zn­(BDP)_2_ and a strong effect that scattered light has on anisotropy measurements. The emission was monitored at 805 nm.

Excitation spectra were recorded for four different excitation–emission polarization combinations, vertical–vertical (VV), horizontal–horizontal (HH), vertical–horizontal (VH) and horizontal-vertical (HV). The anisotropy value, ⟨*r*⟩, was determined according to eq 1, where *I*_XX_ denotes the intensity for a given combination (XX), and the *G*-factor is the polarization-dependent instrument response parameter.^38–40^

1 $$\left\langle r\right\rangle =\frac{I_{VV}-G\times I_{VH}}{I_{VV}+2G\times I_{VH}},where\;G=\frac{I_{HV}}{I_{HH}}$$

Time-resolved fluorescence measurements were performed using a time-correlated single photon counting (TCSPC) system consisting of a picosecond diode laser (PicoQuant), MCP-PMT detector (Hamamatsu R2809U) and a TCSPC board (Becker & Hickl, SPC-730).

### Quantum Chemistry Calculations

Density functional theory (DFT) and time-dependent DFT (TDDFT) calculations were performed using Firefly 8.2.0 ^41^ and Gaussian 16 ^42^ software packages. The results were visualized using Chemcraft 1.7 software. In the final geometry optimizations as well as in TDDFT calculations, performed using Gaussian 16, the solvent effects (toluene) were included using the dielectric continuum model in the solvent model density (SMD) parametrization. The calculations were performed using wB97XD/cc-pVTZ model chemistry. The optimized geometries and TDDFT spectra are reported in the SI (Supporting Information 3).

### Spectral Modeling Using the Frenkel-Holstein Hamiltonian

The electronic absorption spectrum of Zn­(BDP)_2_ was modeled using the Frenkel-Holstein Hamiltonian (eq 2),^43,44^ following the methods described previously.^36,45–47^ According to the model, the potential energy surfaces of the BDP ligands in the ground and excited states are given by shifted harmonic wells with identical curvatures and, therefore, having a common vibrational frequency ω_vib_. The Hamiltonian in the excited-state subspace is expressed as

2 $$H=E_{M}+D+J_{12}\left(|1\rangle \left\langle 2|+|2\right\rangle \langle 1|\right)+\omega_{vib}\sum_{n=1,2}b_{n}^{+}b_{n}+\omega_{vib}\lambda \sum_{n=1,2}\left(b_{n}^{+}+b_{n}+\lambda \right)|n\rangle \langle n|$$

where numbers *n* denote the two BDP ligands (*n* = 1, 2) that are excitonically coupled, and |*n*⟩ represents the electronic state vectors, where ligand *n* is electronically excited, while its neighbor remains in the ground state. The first three terms in eq 2 represent the electronic energy of the system, where *E*_M_ is the 0–0 transition energy for an individual ligand, *D* represents the stabilization energy associated with the nonresonance interaction between the two ligands, and *J*_12_ is the Coulombic coupling between the two ligands, which was estimated in the point dipole approximation

3 $$J_{12}=\frac{1}{4\pi \varepsilon_{op}\varepsilon_{0}}\left(\frac{\left(\mu_{1}\cdot \mu_{2}\right)}{r^{3}}-3\frac{\left(\mu_{1}\cdot r\right)\left(\mu_{2}\cdot r\right)}{r^{5}}\right)$$

In eq 3, **μ**_**1**_ and **μ_2_** are the electronic transition dipole moments for the two BDP ligands, ***r*** is the position vector connecting their centroids, *r* is the distance between the two centroids, and ε_op_ is the (relative) optical dielectric constant.^36,48–50^ Since in Zn­(BDP)_2_ the angles between ***r*** and the two transition dipole moment vectors **μ**_**1**_ and **μ_2_** are 90°, the second term in eq 3 vanishes, and *J*_12_ becomes a function of the magnitudes of **μ**_**1**_ and **μ_2_**, distance *r*, and the dihedral angle (θ) between the ligands.

4 $$J_{12}=\frac{\left|\mu_{1}\right|\left|\mu_{2}\right|\cos\theta }{4\pi \varepsilon_{op}\varepsilon_{0}r^{3}}$$

The last two terms in eq 2 account for the vibrational energy and the local excitonic-vibrational coupling. Here, *b*_*n*_^+^ and *b*_*n*_ are the harmonic oscillator raising and lowering operators with respect to the ground electronic state potential well, ω_vib_λ^2^ is the energy of the nuclear relaxation subsequent to the vertical excitation between the ground and displaced excited well potentials; and λ^2^ is the Huang–Rhys (HR) factor, which can be extracted from the absorption spectrum of the complex with a single ligand, e.g. *mono*-dipyrrinate Zn­(BDP)­Cl.^36,51,52^

The presence of electronic and nuclear degrees of freedom can be directly appreciated by representing the Hamiltonian in eq 2 in the one-particle and two-particle basis set. Hence, to model the spectrum of Zn­(BDP)_2_, we consider one-particle states $|n,\overset{\sim }{v}\rangle$ , where a vibronic excitation involving *ṽ* vibrational quanta in the shifted excited state well resides on chromophore *n* (while the other chromophore remains unexcited), as well as two-particle states $|n,\overset{\sim }{v};n^{\prime },v^{\prime }\rangle$ , where a vibronic excitation resides on *n* and a pure vibrational excitation with *v*' quanta (*v*′ > 0) in the unshifted ground state well resides on $n^{\prime }$ ( $n^{\prime }$ ≠*n*). The eigenfunctions for the system are expanded in the one and two-particle basis set as

$$|\psi_{i}\rangle =\underset{n,\overset{\sim }{v}}{\sum }c_{n,\overset{\sim }{v}}^{\left(i\right)}|n,\overset{\sim }{v}\rangle +\underset{n,\overset{\sim }{v}}{\sum }\underset{n^{\prime },v^{\prime }}{\sum }c_{n,\overset{\sim }{v},n^{\prime },v^{\prime }}^{\left(i\right)}|n,\overset{\sim }{v};n^{\prime },v^{\prime }\rangle$$ 5

The model assumes that the BDP ligands experience negligible molecular orbital overlap in the ground state, such that the Coulombic coupling is the dominant interaction between them, which is consistent with both the experimental X-ray structure data^29^ and DFT calculations. In the ground state, the dihedral angle θ between the two ligand mean–square planes is 52.66° (wB97XD/cc-pVTZ), resulting in a non-negligible dipole–dipole interaction. In the absence of vibronic coupling, the two exciton states would be the symmetric (sym) state, 2^–1/2^(|1⟩ + |2⟩), with transition dipole moment 2^–1/2^(**μ**_**1**_ + **μ**_**2**_), and the antisymmetric (ant) state, 2^–1/2^(|1⟩-|2⟩) with transition dipole moment, 2^–1/2^(**μ**_**1**_ – **μ**_**2**_). Here, symmetry is defined with respect to the *C*_2_ axis shown in Figure 1b. Since *J*_12_ > 0, the symmetric state with higher oscillator strength is the higher-energy state. In the limit of θ = 0°, the upper state consumes all of the oscillator strength, leaving a completely dark lower-energy state, as is characteristic of an H-dimer.

The transition dipole moment of a single BDP ligand in Zn­(BDP)_2_ was approximated by the transition dipole moment calculated for monodipyrrinate Zn­(BDP)­Cl·Pyr using TDDFT, from which we estimated the initial value of the excitonic coupling between the BDP ligands (*J*_12_) using eq 3 and the geometric parameters, angle θ and distance *r*, obtained from the ground state DFT calculations of Zn­(BDP)_2_. We used ε_op_ = 2.23, which is the optical dielectric constant for toluene at 650 nm. The distance *r* was defined as the distance between the centroids of the two dipyrrin ligands, where each centroid was calculated for 19 atoms comprising the basic dipyrrin skeleton and the two fused benzo-rings, omitting the *meso*-aryl substituent and ester groups. Then, θ and *r*, and consequently *J*_12_, were allowed to vary in order to determine the best fit to the experimental absorption spectrum. Varying θ and *r* was justified by the fact that the calculated geometries do not account for explicit solvent effects and the fact that the point dipole formalism is only an approximation.

### Electrochemistry

Cyclic voltammetry (CV) measurements were carried out at room temperature using solutions of Zn­(BDP)_2_ in dichloromethane (CH_2_Cl_2_) containing 0.1 M tetra-*n*-butylammonium perchlorate (TBAP), using a potentiostat/galvanostat (EG&G Princeton Applied Research, model 173). A three-electrode system was used, which consisted of a glassy carbon working electrode, a platinum wire auxiliary electrode, and a saturated calomel electrode (SCE) as the reference electrode. The SCE was separated from the bulk of the solution by a salt bridge of low porosity which contained the solvent/supporting electrolyte mixture. High purity N_2_ was used to deoxygenate the solution, and the solution was kept under N_2_ during the experiment. CH_2_Cl_2_ (≥99.8%, EMD Chemicals Inc.) and TBAP (≥99.0%, Sigma-Aldrich co.) were used as received.

### Ultrafast Nonlinear Spectroscopy

Femtosecond transient absorption spectroscopy (fsTA) and two-dimensional electronic spectroscopy (2DES) measurements were performed on samples of Zn­(BDP)_2_ and Zn­(BDP)­Cl. The concentrations of samples were such that the absorbances at the absorption maxima were 0.25–0.30 OD in an optical cell with a 1 mm path length (STARNA, 21-Q-1). UV/Vis absorption spectra were recorded before and after ultrafast measurements to confirm that the samples were stable over the time frame of the measurements.

The setup for fsTA spectroscopy has been described previously.^53,54^ Briefly, the pump and probe pulse pair were generated from the output of a commercial Ti/sapphire laser (Coherent Libra, 4 W, 100 fs pulses with a 1 kHz repetition rate, λ_max_ = 800 nm). A portion of the laser output was directed into a home-built noncollinear optical parametric amplifier (NOPA) to generate pump pulses centered at 595 nm (504 THz) with the fwhm of 27 THz. The NOPA output was compressed with a single grating and prism compressor.^55^ The compressed NOPA output was characterized by second harmonic generation frequency resolved optical gating (SHG-FROG) with a 100 μm BBO crystal, resulting in pulses with a temporal width of 27 fs, as reported in the SI (Supporting Information 1).^56^ The white light continuum, acting as a probe, was generated by focusing another portion of the 800 nm laser output into a 3 mm-thick sapphire crystal. The spectrum of the generated white light continuum spans the range of 450–750 nm. The pump and probe pulses used for the fsTA measurements along with the absorption spectra of Zn­(BDP)_2_ and Zn­(BDP)Cl are shown in SI 1. The NOPA pump pulse energy at the sample stage was 37 nJ, and the probe pulse energy was below 15 nJ. The pump and probe pulses were linearly polarized and set at the magic angle, 54.7°, with respect to one-another. The delay time between the pump and probe pulses, *t*_2_, was scanned from −10 ps to 1.2 ns in varying step sizes (0.1 ps steps from −0.2 to 5 ps, 0.5 ps steps from 5 to 10 ps, 1 ps steps from 10 to 600 ps, 2 ps steps from 600 to 800 ps, 5 ps steps from 800 ps to 1 ns and 10 ps steps from 1 to 1.2 ns) by a computer-controlled translation stage (Newport ILS250 cm^3^, XPS Q8). Throughout the text we refer to the *t*_2_ time as the waiting time. An optical chopper was placed in the pump beam path to chop the beam at 500 Hz. The probe light transmitted through the sample (with the pump on/off) was spectrally resolved with a spectrometer (Andor, Shamrock 500i) and recorded with a CCD camera (Andor Newton, EMCCD: DU970P-FI). Changes in the optical density (ΔOD) were obtained from the collected spectra and reported as a function of *t*_2_ waiting time. The reported transient spectra are averages of 500 spectra for each delay time *t*_2_.

The details of the 2DES setup were reported previously.^57^ In brief, 2DES experiments were conducted using a pulse shaper in the pump–probe geometry, so that purely absorptive spectra were obtained.^58–60^ Visible pulses centered at 625 nm (480 THz) with the fwhm of 74 THz were generated with a home-built NOPA. A portion of the NOPA output was directed into a programmable acousto-optic dispersive filter (Dazzler, Fastlite) to generate two pump pulses centered at 625 nm (480 THz) with a fwhm of 42 THz which are separated by a time delay referred to as *t*_1_. A Grism compressor (Fastlite) was placed before the Dazzler to precompensate for the dispersion associated with the Dazzler. The Dazzler was used to scan the *t*_1_ time delay between the two pump pulses from −50 to 0.1 fs in 256 evenly spaced steps. For each *t*_1_ time (the time between the two pump pulses), a phase cycling scheme *S*(0,0) – *S*(0,π) + *S*(π,π) – *S*(π,0) was used to remove the background and scatter, where the phase of pump pulse 1, ϕ_1_, and the phase of pump pulse 2, ϕ_2_, are indicated as S­(ϕ_1_,ϕ_2_).

The probe pulse was generated from another portion of the NOPA output. It was centered at 625 nm (480 THz) with a fwhm of 74 THz and delayed by a series of waiting times (*t*_2_). The 2DES spectra were obtained using a partially rotating frame with a frame rotation frequency of 350 THz.^58^ The pump pulses were characterized by sum-frequency generation cross-correlation FROG (SFG-XFROG) with the probe pulse as a reference pulse.^61^ The duration of the pump pulse was 18 fs (Supporting Information 1). The pump pulses were linearly polarized at the magic angle of 54.7° with respect to the probe pulse, where the polarization of the probe pulse was set to be parallel to the optical table. The power of the incident pulses was measured to be 19 nJ before the sample cell.

### Nanosecond Transient Absorption Spectroscopy (nsTA)

nsTA measurements were performed using a home-built setup, which included a 355 nm pump pulse from a frequency-tripled Nd/YAG laser (Quanta-Ray DCR-1A, 15 mW, 10 ns pulses with 10 Hz repetition rate) and a white-light probe pulse generated by a flash lamp (Hamamatsu, 2 μs pulses). The light transmitted through the sample was spectrally resolved by a monochromator (SPEX Inc.) and registered with a diode-array detector (Princeton Instruments, DIDA-512). In addition, the time evolution of the spectral features at selected wavelengths (550, 600, 625, and 680 nm) was measured using a commercial nsTA Instruments (Magnitude Instruments, enVISion). The measurements were conducted using aerated samples as well as samples deoxygenated by N_2_ purging.

## Results and Discussion

### Linear Absorption and Fluorescence Measurements

The equilibria between the *mono*-dipyrrinate, Zn­(BDP)­X, and the homoleptic *bis*-dipyrrinate, Zn­(BDP)_2_, as well as the associated linear absorption and emission spectra were described previously.^29^ Here we extended our characterization by measurements in solvents of different polarity in view of the possible involvement of polar or charge transfer states in the excitation dynamics. The UV–vis absorption and fluorescence data are summarized in Table 1.

**Table 1:** Photophysical Properties of Zn­(BDP)­Cl and Zn­(BDP)_2_ at 23 °C

compound	solvent	ε^a^	λ_max_ abs. (nm)	λ_max_ fluo (nm)	ϕ^b^	τ (ps)^c^
Zn(BDP)Cl	DMF	36.7	634	640	6.5 × 10^–1^	1670
Zn(BDP)_2_	cHex	2.02	595	802	7.1 × 10^–3^	870
	TolH	2.38	597	795	5.0 × 10^–3^	678
	Et_2_O	4.33	593	790	3.3 × 10^–3^	490
	MeCN	37.5	590			

a Static dielectric constant.

b Fluorescence quantum yield measured against fluorescence of Oxazine-1 in ethanol (ϕ = 0.15)^62^.

c Fluorescence decay time.

The absorption spectra of Zn­(BDP)_2_ in four different solvents, MeCN, Et_2_O, TolH, and cHex, are shown in Figure 2a along with the spectrum of Zn­(BDP)Cl in DMF (gray line). The latter has a visible absorption band with the maximum at 634 nm and a shoulder extending to the blue up to 500 nm, presumably arising from a vibronic progression. A nearly identical spectrum (λ_max_ = 637 nm) was recorded in pyridine previously.^29^ Similar features are present in the spectra of BODIPY and other metallo-dipyrrins.^1,2,4–6,10^

**Figure 2:**
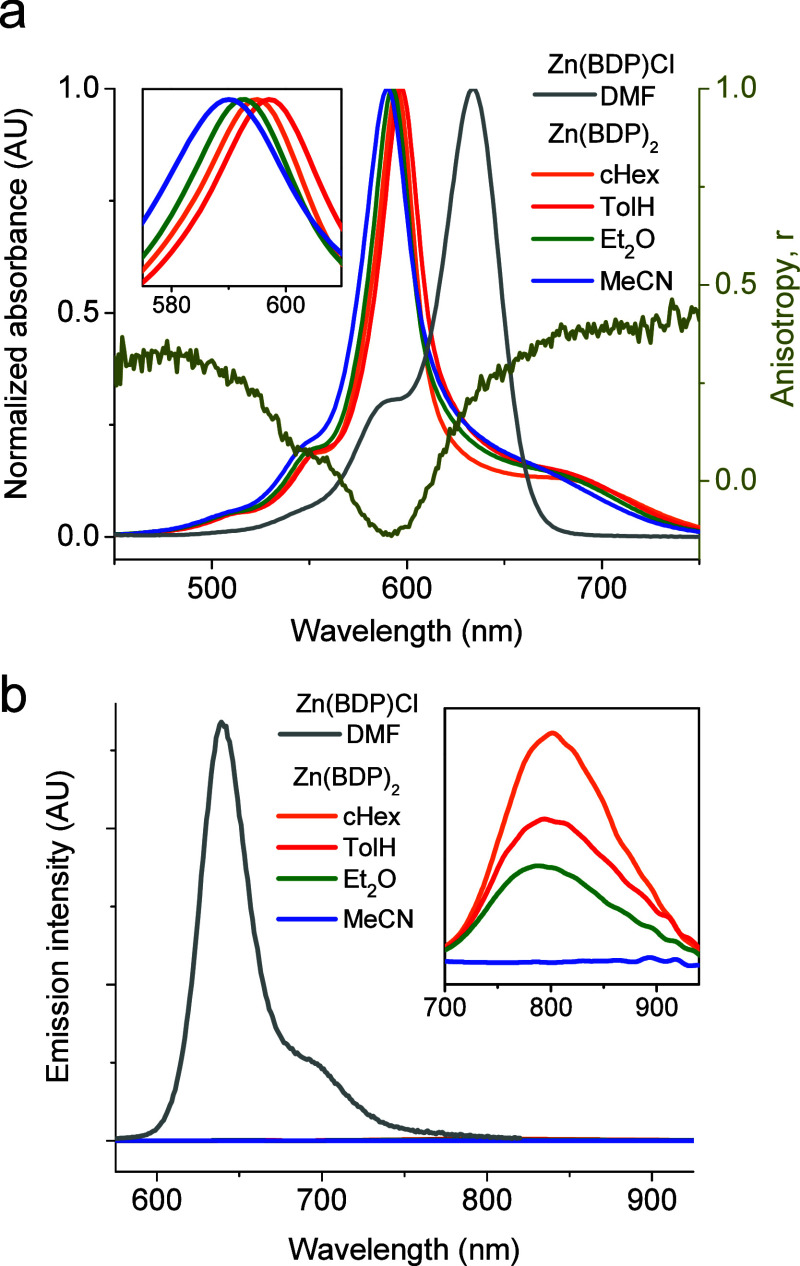
(a) Optical absorption spectra of Zn­(BDP)_2_ in cHex (orange), TolH (red), Et_2_O (green), and MeCN (blue) and of Zn­(BDP)­Cl in DMF (gray). Fluorescence excitation anisotropy spectrum of Zn­(BDP)_2_ (dark yellow; see Methods for details) is plotted against the right axis. (b) Fluorescence spectra of Zn­(BDP)_2_ (λ_ex_ = 597 nm) and of Zn­(BDP)Cl (λ_ex_ = 550 nm). The integrals under the fluorescence spectra are proportional to the emission quantum yields.

The spectrum of Zn­(BDP)_2_ is more complicated, having the maximum at 592–600 nm, depending on the solvent, a blue shoulder in the 500–550 nm region, resembling a vibronic progression, and a broad featureless red shoulder extending beyond 700 nm (Figure 2a).

Previously, changes in the absorption spectra of Zn­(BDP)_2_, compared to Zn­(BDP)­Cl, have been attributed to the formation of excitonic states, where the close proximity of the BDP ligands and their nonorthogonal orientation could lead to a non-negligible coupling between the transition dipole moments.^29^ Excitonically coupled dimeric systems are expected to exhibit absorption spectral features similar to those seen in the case of Zn­(BDP)_2_, where the most intense blue-shifted transition is typically assigned to the first vibronic transition and the red-shifted shoulder to the purely excitonic transitions.^63,64^

According to the molecular exciton model,^34^ the transition to the lower excitonic state in the system consisting of two chromophores (a dimer) in oblique orientation to one-another is characterized by a low oscillator strength. Consequently, the decay from that state has a low radiative rate constant, and the emission is outcompeted by internal conversion and/or intersystem crossing, leading to an increase in the triplet yield. Indeed, while Zn­(BDP)­Cl exhibits strong fluorescence (λ_max_ = 640 nm, ϕ_fl_ = 0.65 in DMF)^29^ (Figure 2b), the emission of Zn­(BDP)_2_ is ∼100 times weaker, and the emission band has a broad featureless shape, resembling the red shoulder in the absorption spectrum. Fluorescence excitation spectra confirmed the identity of the emitting species (Supporting Information 2).

Both the fluorescence quantum yield and lifetime of Zn­(BDP)_2_ were found to be solvent-dependent, decreasing with the solvent polarity (increasing static dielectric constant ε) (Table 1). In the case of the most polar solvent tested (MeCN, ε = 37.5), no emission could be detected at all. The dependence of the emission yield and rate constant on the solvent suggests involvement of a polar state(s) in the relaxation pathway. However, if the fluorescence were to originate from such a polar state, the emission band would be expected to shift bathochromically, as the polar state would become more stabilized in more polar solvents. Instead, the emission maximum of Zn­(BDP)_2_ shifts to the blue with an increase in ε (Figure 2b, Table 1), suggesting that the fluorescent state is not polar, and yet its decay is influenced by a state sensitive to the solvent polarity. A possible mechanism explaining these observations is discussed below.

Since excitonic states are associated with orthogonal transition dipole moments^38^ (see Experimental: Frenkel-Holstein Hamiltonian), polarization dependent fluorescence measurements can be used to characterize excitonic transitions, provided the time scale for molecular tumbling (reorientation) is long compared to the emission lifetime. This avoids the need for orientational averaging. We measured fluorescence excitation spectra of Zn­(BDP)_2_ dissolved in a solid polymer film (polymethyldisiloxane, PDMA) to extract the fluorescence anisotropy (Figure 2a, dark yellow line). Excitation to the upper excitonic state using linearly polarized light results in emission polarized in the perpendicular direction (assuming that it originates in the lower excitonic state), while excitation to the lower excitonic state should produce emission polarized in the same direction.^38^ Indeed, the anisotropy value, ⟨*r*⟩ (eq 1), was found to be ∼ −0.2 when exciting at 597 nm (the vibronic transition associated with the upper excitonic state) and ∼0.4 when exciting at 680 nm (the lower excitonic state). It is worth noting that a transition to a polar/CT state could also be polarized orthogonally to the transition to the upper excitonic state; and hence if the fluorescence were to originate from such a polar state, the anisotropy spectrum could look similar to the one shown in Figure 2a. However, as discussed above, fluorescence from a polar state would be inconsistent with the observed solvent dependence of the emission spectra.

Overall, linear absorption and emission measurements were found to be consistent with the presence of excitonic coupling in the Zn­(BDP)_2_ complex. To further characterize the corresponding transitions, spectral modeling was performed using a Frenkel-Holstein Hamiltonian as described below.

### Modeling of the Initial Excited States in Zn­(BDP)_2_

The previously published X-ray crystallographic structure of a Zn *bis*-dibenzodipyrrin revealed close proximity of the BDP ligands and their nonorthogonal orientation.^29^ Ground state DFT calculations confirmed that the observed nonorthogonality is an intrinsic structural feature of Zn *bis*-2,2′-alkoxycarbonyldipyrrinates,^30^ as opposed to being caused by crystal packing forces. Here we performed DFT and TDDFT calculations of Zn­(BDP)_2_ to confirm the nonorthogonal geometry in the ground state as well as in the excited state (see Supporting Information 3).

The TDDFT spectra of Zn­(BDP)_2_ revealed qualitatively correct distribution of the oscillator strengths between the transitions (Supporting Information 3); however, the fine vibronic structure, which is required for interpretation of the experimental spectrum, could not be obtained from these calculations. For this reason, we used the Frenkel-Holstein Hamiltonian (eq 2) and the basis consisting of one-particle vibronic and two-particle vibronic/vibrational states (see Experimental, eq 5).^36,43,44,46,47,51,65,66^ The modeled absorption spectrum and a proposed energy level diagram are shown in Figure 3.

**Figure 3:**
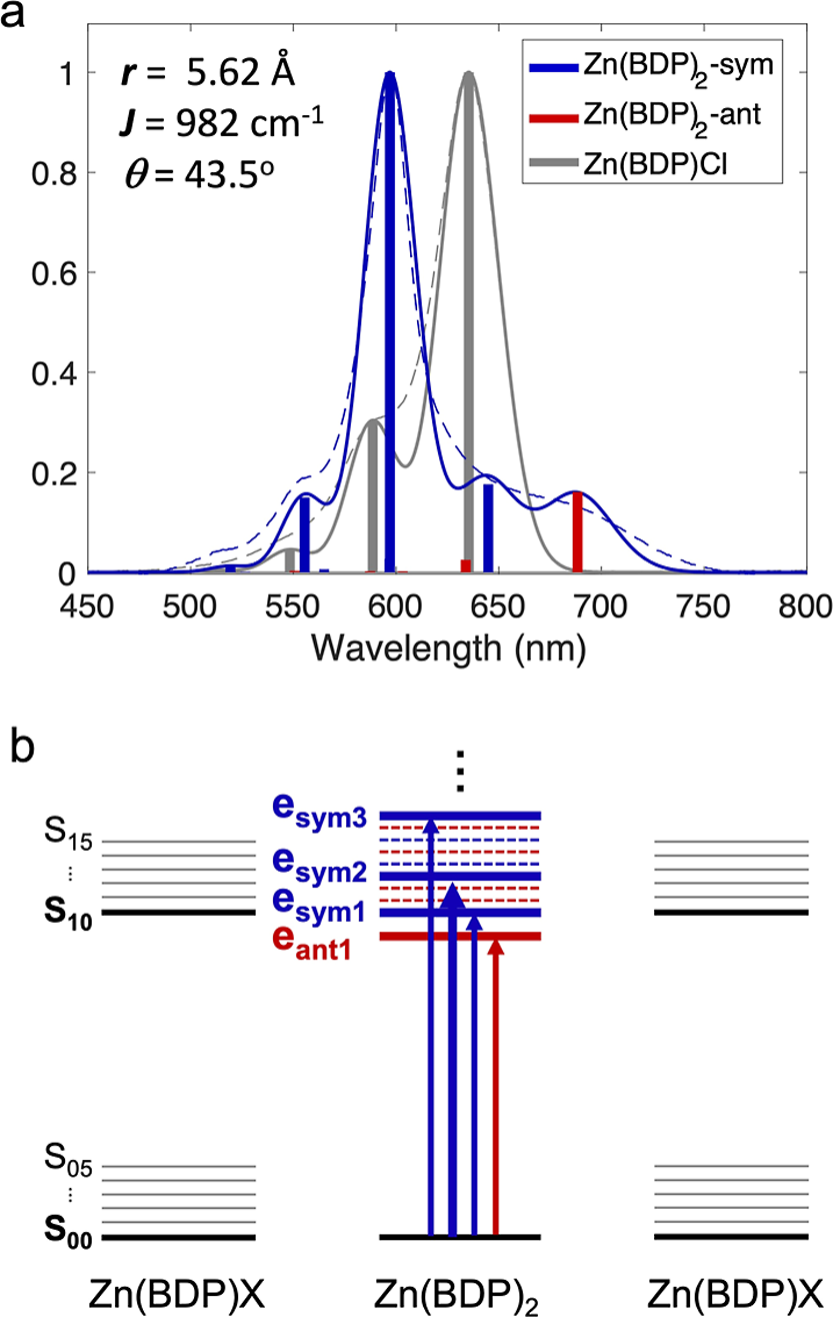
(a) The experimental (dashed lines) and simulated (solid lines) absorption spectra of Zn­(BDP)­X (gray) and Zn­(BDP)_2_ (blue). Symmetric (sym) and antisymmetric (ant) excitonic states are color-coded in blue and red, respectively. The simulated spectra (vertical lines) are broadened by Gaussians, fwhm = 500 cm^–1^ (15 THz), to facilitate visual comparison with the experimental spectra. The parameters used to simulate the spectra were: *r* = 5.62 Å, θ = 43.5°, (giving a *J*_12_ = 982 cm^–1^), ε_op_ = 2.23, |**μ**| = 10.32 D, HR factor = 0.28, ω_vib_ = 1250 cm^–1^, *E*_M_ = 15,738 cm^–1^, *D* = 335 cm^–1^. (b) Energy level diagram corresponding to the transitions associated with the spectra shown in (a) where the solid red and blue lines associated with Zn­(BDP)_2_ correspond to transitions with larger oscillator strengths and the dashed lines correspond to weaker or dark transitions. Oscillator strengths and energies are reported in the Supporting Information 4.

The spectrum of Zn­(BDP)Cl was modeled first in order to extract the Huang–Rhys (HR) factor (λ^2^) and the effective Franck–Condon mode, ω_vib_, which were found to be 0.28 and 1250 cm^–1^, respectively. The experimental spectrum of Zn­(BDP)Cl could be well-approximated using six vibronic states where the main features are assigned to the S_10_, S_11_, and S_12_ transitions (Figure 3). The agreement between the simulated and experimental spectra suggests that the determined values are adequate for modeling the Zn­(BDP)_2_ complex.

The electronic coupling between the two ligands, *J*_12_, in Zn­(BDP)_2_ was estimated using the point-dipole approximation (eq 3), where the transition dipole moments for the individual ligands (**μ_1_** and **μ_2_**) were assumed to have the value of 10.32 D, computed using TDDFT for Zn­(BDP)­Cl·Pyr (see Methods, Materials and Supporting Information 3).

The values for θ and *r* were 52.66° and 5.97 Å, respectively. The corresponding value of *J*_12_ was calculated to be ∼685 cm^–1^, and the absorption spectrum simulated using the above parameters is shown in Supporting Information 4. The dihedral angle θ, distance *r* and, consequently, the coupling constant *J*_12_, were then adjusted, using the fminsearch function in Matlab, to obtain a better fit to the experimental spectrum. The resulting optimized values were θ = 43.5°, *r* = 5.62 Å and *J*_12_ = 982 cm^–1^. The corresponding spectrum is shown in Figure 3a.

The basis set was constructed considering 6 vibrational states (*v* = 0–5) for each electronic state of each ligand using the one-particle and two-particle basis set (See Figure S13 for comparison with the one-particle basis set.). The 12 lowest energy excited states resulting from diagonalizing the Hamiltonian for Zn­(BDP)_2_ (Supporting Information 4) were designated as symmetric (sym) or antisymmetric (ant) with respect to the *C*_2_ symmetry operation (Figure 1). The normalized spectra are shown in Figure 3a. It was found that 4 excitonic states, corresponding to the lines with the maxima at 687 nm, 644 nm, 597 and 555 nm, dominate the spectral profile. The transition dipole moment corresponding to the lowest energy state *e*_ant1_ (687 nm) scales as |**μ**_**1**_ – **μ**_**2**_|. The transition dipole moments for the states *e*_sym1_, *e*_sym2_, and *e*_sym3_ (644 nm, 597 and 555 nm) are proportional to |**μ**_**1**_ + **μ_2_** |. The absorption maximum at 597 nm corresponds to *e*_sym2_, while the red shoulder has contributions corresponding to *e*_sym1_ and *e*_ant1_.

The spectral simulation (Figure 3) is in agreement with the results of the fluorescence anisotropy measurements and, in general, with the assignment of the initially populated excited states of Zn­(BDP)_2_ as molecular excitonic states. Furthermore, the simulations provide additional information about the fine vibronic structure of the observed transitions and define a theoretical framework for the assignment of these transitions based on 2DES and TA characterization (see below).

### Two-Dimensional Electronic Spectroscopy (2DES)

2DES is a nonlinear spectroscopic technique that is well suited for the investigation of excitonically coupled systems.^67–70^ In a 2DES spectrum, peaks that lie along the diagonal correspond to the transitions observed in the conventional linear absorption spectrum, albeit their intensities scale as |**μ**|^4^ as opposed to |**μ**|^2^ in linear spectra. The presence of cross-peaks at early waiting times in 2DES spectra indicate that the transitions corresponding to the diagonal peaks are strongly coupled. The intensities of the cross-peaks scale as |**μ_A_** |^2^|**μ_B_** |^2^ where **μ**_**A**_ and **μ**_**B**_ are the transition dipole moments corresponding to the diagonal transitions. If one of the transitions is weakly allowed, but strongly coupled to a transition with a large **μ**, the cross-peak at an early time could be useful for extracting information about the weaker transition.^71^ We used this aspect of 2DES to further confirm the assignment of the transitions of Zn­(BDP)_2_ to excitonic states.

2DES spectra of Zn­(BDP)_2_ in TolH and Zn­(BDP)Cl in pyridine at an early waiting time are shown in Figure 4 (see Supporting Information 5 for the 2DES spectra at later waiting times). As expected, the 2DES spectrum of Zn­(BDP)­Cl exhibits one main peak along the diagonal with λ_max_∼630 nm. The main peak has contributions from the S_00_ → S_10_ transition and Franck–Condon (FC) active modes that lie within the bandwidth of the incoming pump laser pulse (see Supporting Information 1 for the pulse spectra). At *t*_2_ = 37 fs, the spectral profile of the main peak is elongated along the diagonal due to inhomogeneous broadening. As the waiting time increases the spectral profile becomes box-like in shape as the inhomogeneity decays and vibrational relaxation occurs among the FC modes (Figure S14). Overall, the 2DES spectrum of Zn­(BDP)­Cl resembles the spectra of monomeric BODIPY systems studied previously.^54^

**Figure 4:**
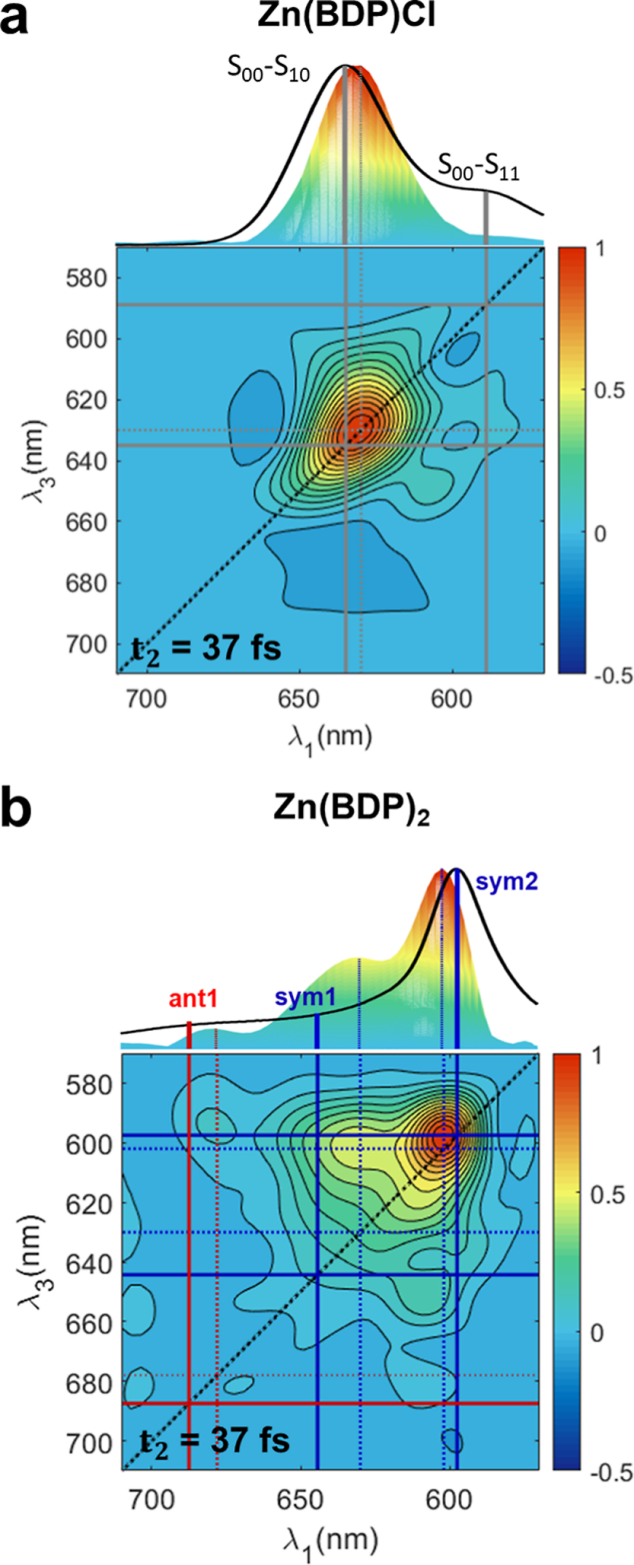
2DES spectra of (a) Zn­(BDP)Cl in pyridine and (b) Zn­(BDP)_2_ in TolH at a waiting time *t*_2_ = 37 fs are plotted where the diagonal is noted as a dashed black line. The upper panels above the 2DES spectra display the linear absorption spectra as solid black lines along with the side view of the 2DES spectra projected on the (λ_1_, *z*) plane. For Zn­(BDP)Cl (a), the maxima of the S_00_–S_10_ and S_00_–S_11_ transitions from the model are indicated as solid gray sticks in the linear spectrum and the wavelengths associated with these transitions are noted with solid gray lines superposed on the 2DES spectrum. The maximum wavelength associated with the main peak in the projection of the 2DES spectrum is noted with a dashed gray line. For Zn­(BDP)_2_ (b), the transitions predicted by the Frenkel-Holstein model (Figure 3) are noted as solid red and blue sticks in the linear spectrum and as solid lines superposed on the 2DES spectrum. The red and blue dotted lines indicate the maxima in the (λ_1_, *z*)-projections of the 2DES spectrum and are noted as horizontal and vertical dashed lines superposed on the 2DES spectrum.

The key feature of the 2DES spectrum of Zn­(BDP)_2_ (Figure 4b) that is central to the present study is the presence of cross-peaks at (λ_1_, λ_3_) = (602 nm, 630 nm), (602 nm, 678 nm), (630 nm, 602 nm) and (678 nm, 602 nm), which reveal strong coupling between the corresponding diagonal transitions. We note that the peaks in the 2DES spectra are slightly shifted compared to those in the linear spectrum, which is due to the fact that the 2DES spectral response is affected by the spectra of the incoming laser pulses (see Supporting Information 1). An important detail, which is clearly observable in the 2DES spectrum, but could not be resolved in the linear spectrum, is the presence of the low energy excitonic transitions, *e*_ant1_ and *e*_sym1_ (Figure 3). The cross peak features of the 2DES spectrum further confirm the excitonic nature of the transitions due to the close proximity of the BDP ligands and their nonorthogonal orientation in Zn­(BDP)_2_.^29^ The presence of the cross peaks and their energy levels in the 2DES spectra are in agreement with the assignment based on the modeling with the Frenkel-Holstein Hamiltonian, and confirm that the initial states are excitonically coupled states.

### Excited States Evolution

fsTA in combination with global analysis,^72,73^ nsTA, and fluorescence time-resolved spectroscopies were used to study the evolution of the initial excitonic states. The fsTA spectra of Zn­(BDP)Cl and Zn­(BDP)_2_ are displayed in Figure 5 along with the corresponding kinetic traces at selected wavelengths.

**Figure 5:**
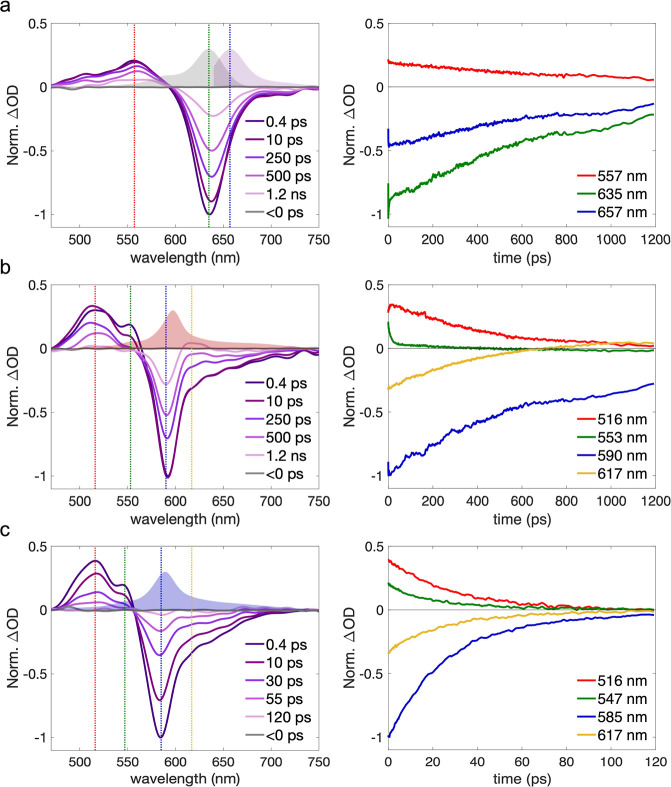
fsTA spectra and temporal ΔOD traces at selected wavelengths for (a) Zn­(BDP)Cl in pyridine, (b) Zn­(BDP)_2_ in TolH, and (c) Zn­(BDP)_2_ in MeCN. The linear absorption and fluorescence (for Zn­(BDP)­Cl) spectra are illustrated as shaded areas.

The spectra of Zn­(BDP)Cl (Figure 5a) exhibit bands associated with excited state absorption (ESA), ground state bleach (GSB), and stimulated emission (SE) with the maxima at 557 nm, 635 and 657 nm, respectively. The GSB and SE maxima were assigned based on the linear absorption and emission spectra (Figure 2). The ΔOD kinetic traces show that the GSB, SE, and ESA bands decay on similar time scales, which is consistent with the direct S_1_ → S_0_ deactivation. Global analysis confirmed the presence of a single decay-associated spectrum (DAS) that evolved with the time constant of 880 ps (see Supporting Information 7 for details), which is similar to the fluorescence lifetime measured by TCSPC and reported in Table 1 (see Supporting Information 11).

The early fsTA spectra of Zn­(BDP)_2_ are similar across all solvents (see Figure 5b,c for TolH, MeCN and SI 6 for Et_2_O, cHex). The dominant feature is a negative band centered at ∼600 nm with a shoulder extending beyond 700 nm, matching the absorption profile in the UV/vis. This band is primarily due to the GSB. We note that the SE (near 780 nm) is not readily observable, which is expected given the low extinction coefficient associated with this band in the linear absorption spectrum. The positive feature to the blue of the GSB band, with the main peak at 516 nm and an additional feature at 553 nm, is due to the ESA associated with the excitonic states. The evolution of this spectral feature was found to be dependent on the solvent polarity.

In MeCN, the ESA feature at 516 nm as well as all other spectral features decay on similar time scales. Global analysis revealed a single DAS with a decay time constant of 28 ps. However, for less polar solvents, different spectral features evolve with different rates that are dependent on the solvents’ dielectric constants (Figure 5b). The ESA at 553 nm first decays rapidly in synchrony with a growth of the peak at 516 nm. However, the growth does not appear to be as prominent as the decay, which could be a combined effect of two overlapping ESA features. It is evident from the traces (Figure 5) that the growth and decay occur faster in solvents with higher polarity, being barely resolvable in Et_2_O, and not resolvable at all in MeCN.

After the initial fast phase, the ΔOD at 516 nm decays over hundreds of picoseconds, again with the rate dependent on the solvent polarity, while a new ESA feature emerges at 617 nm. Both the GSB and the band near 617 nm do not completely decay by 1.2 ns (the upper time limit of our fsTA system), persisting over microseconds (see the discussion of the nsTA data below).

Singular value decomposition (SVD) analysis and extracted DAS revealed that the spectral evolution of Zn­(BDP)_2_ in TolH and other nonpolar solvents could be adequately described using three components (see Supporting Information 7 for details). To determine the time scales associated with the evolution of these components we extracted evolution-associated spectra (EAS) and determined that a sequential model, A → B → C, produces the best fit to the experimental fsTA spectra. The extracted EAS and temporal traces are shown in Figure 6.

**Figure 6:**
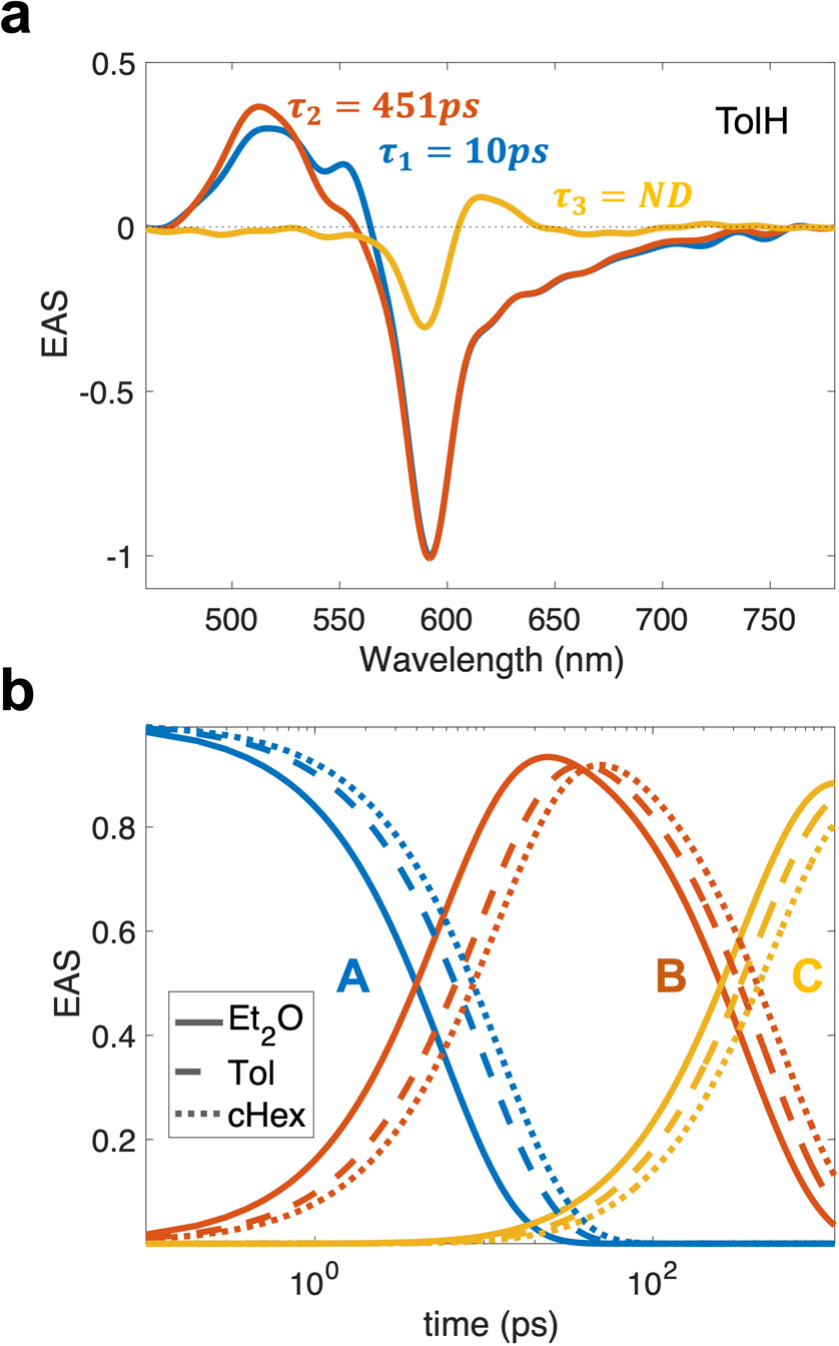
(a) EAS extracted from fsTA of Zn­(BDP)_2_ in TolH. ND refers to “nondecaying” and denotes the lifetime of the component *C*, τ_3_. (b) Evolution traces associated with each EAS (A (blue), B (orange), and C (yellow)) in Et_2_O (solid line), TolH (dashed line), and cHex (dotted line).

EAS A has a large negative band spanning across the ∼600–700 nm range and a smaller negative band at ∼550 nm. These bands resemble the inverted linear absorption spectrum (Figure 2), and hence they were assigned to the GSB due to the population of the initial excited states. In addition, there are two positive features, at 516 and 533 nm, that result from the ESA associated with the initial excited states.

EAS A evolves over several picoseconds in a solvent-dependent fashion forming EAS B, which has a positive ESA feature with a peak at ∼510 nm. The negative peaks in EAS A and B fully coincide, indicating that the ground state population does not change during the first few picoseconds, as the excited state population transitions between states A and B (A → B). After reaching its maximum, EAS B evolves further over several hundred picoseconds, again in a solvent-dependent manner, transforming into a long-lived EAS C, which has a new ESA feature near 617 nm. The solvent dependent time constants associated with the evolution of the EAS are summarized in Table 2.

**Table 2:** Time Constants Extracted from the Analyses of EAS (cHex, TolH, Et_2_O) and DAS (MeCN) of Zn­(BDP)_2_ ^a^

solvent	ε	extracted time constants	
		τ_1_ (ps)	τ_2_ (ps)
cHex	2.02	14 ± 1.5	633 ± 114
TolH	2.38	10 ± 0.5	451 ± 1
Et_2_O	4.33	5 ± 0.6	367 ± 14
MeCN	37.5	28 ± 0.9	

a The time constants for a given solvent are an average of three separate measurements and the error bars represent the standard deviation.

Combining the results from the analysis of the fsTA with the fluorescence data, nsTA, 2DES, and electrochemical measurements (vide infra), a plausible description of the photoinduced dynamics in Zn­(BDP)_2_ emerges, which reconciles the earlier evidence of exciton coupling in Zn *bis*-dibenzodipyrrins^29,30^ with possible involvement of charge transfer intermediates, proposed for nonextended Zn *bis*-dipyrrins.^22,32,33^ An energy level diagram summarizing the pathways is shown in Figure 7.

**Figure 7:**
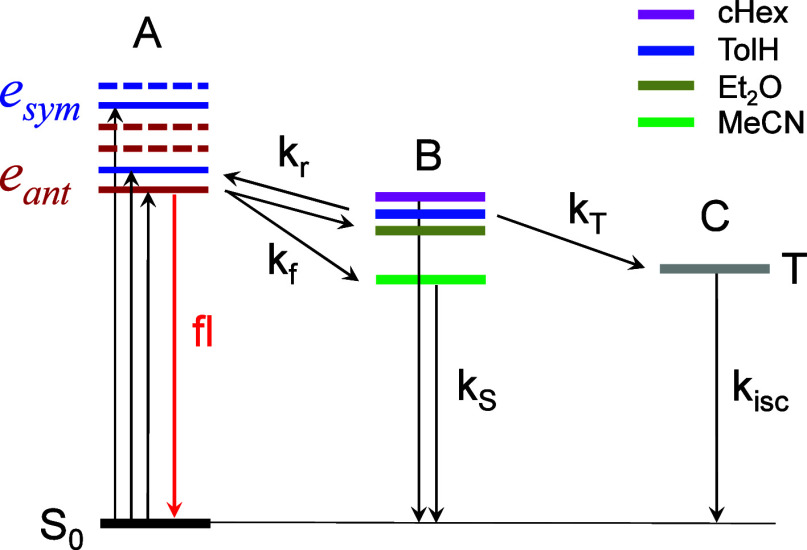
Energy level diagram showing the population dynamics after photoexcitation of Zn­(BDP)_2_.

The linear absorption, fluorescence anisotropy, and 2DES data point to the existence of excitonic coupling in Zn­(BDP)_2_. Furthermore, the Frenkel-Holstein model is able to predict the experimental spectra of Zn­(BDP)_2_ with good accuracy. TDDFT calculations also support the assignment of the initial states to excitonic states (Supporting Information 3). Collectively, these results suggest that EAS A (both GSB and ESA) reflects the initial transitions involving Frenkel excitonic states, which are referred to collectively as state A.

Comparing EAS A to EAS B, we assign the second state in the pathway (state B) to the ESA feature that grows in at 516 nm, overlapping with the ESA of the initial excitonic states. An important feature of state B is the dependence of its rise time on the solvent polarity (Figure 6 and Table 2), which suggests that state B could be polar itself, resembling SBCT states proposed for Zn complexes of nonextended dipyrrins.^22,27,32,33^ Since polar states are stabilized in polar solvents, the thermodynamic driving force for the transition A → B (*k*_f_, Figure 7) should increase with an increase in the solvent dielectric constant ε, provided the energy of the initial state A remains unchanged. The rates of the decay of A and rise of B, which correspond to the initial fast phases in the kinetic traces at 553 and 516 nm (Figure 5), indeed increase with the solvent polarity, cHex → TolH → Et_2_O, which is consistent with faster charge separation at higher driving forces |ΔG_CS_|.

Consistent with previous work on photoinduced charge transfer states in general^74^ and for Zn dipyrrins specifically,^22^ we estimate the driving force for charge separation, Δ*G*_CS_, using the following equation:^40,74,75^ Δ*G*_CS_=(*E*_ox_–*E*_red_)-E*-*w*, where *E*_ox_ and *E*_red_ are the oxidation and reduction potentials of Zn­(BDP)_2_, E* is the energy of the parent excited state, and *w* is the electrostatic work term. Energy E* was taken as that of the lower exciton state *e*_ant1_, E*∼1.79 eV, assuming that the charge separation would occur after the ultrafast *e*_sym_ → *e*_ant_ internal conversion. The redox potentials, determined by cyclic voltammetry vs SCE in DCM (Supporting Information 8), were found to be *E*_1/2_ = −1.05 eV for reduction and *E*_1/2_ = 0.79 eV for oxidation of Zn­(BDP)_2_. To facilitate comparison with previous work,^22,32,74^ we omit the electrostatic term, *w*, which is presumably small in nonpolar solvents^74^ and find Δ*G*_CS_ to be ∼0.05 eV. This value of Δ*G*_CS_ is negligible compared to other Zn dipyrrin systems (Δ*G*_CS_ ranges from 0.19 to 0.28)^22^ and systems that undergo SBCT (Δ*G*_CS_ ranges from −0.24 to 0.35).^74^ Given the comparison with previous systems charge separation could be efficient in the Zn­(BDP)_2_ complexes studied here.

Another plausible scenario is that state B is not a charge-separated state (BDP^+•^-Zn-BDP^–•^), but rather an excimer-like state, i.e. a superposition of a Frenkel excitonic state and a charge-transfer excitonic (CTE) state, where the CTE component increases as the molecule relaxes along the excited state potential energy surface, and its geometry changes to favor stronger short-range electronic coupling between the BDP ligands. Unlike Frenkel excitons (FE), which are superpositions of excitations residing on individual chromophores, 2^–1/2^(|BDP*-Zn-BDP⟩ ± |BDP-Zn-BDP*⟩), charge transfer excitons (CTE) are superpositions of CT states resulting from electron transfer from one chromophore to another, 2^–1/2^(|BDP^•+^-Zn-BDP^•‑^⟩ ± |BDP^•‑^-Zn-BDP^•+^⟩).^36,37^ In the context of our discussion, one important distinction is that unlike SBCT states, FE and/or symmetric CTE states do not possess permanent dipole moments. As will be noted below, our assignment of state B to an excimer-like state is consistent with the fluorescence measurements.

The decay of state B occurs over hundreds of picoseconds, and the decay time constant is solvent-dependent (τ_2_, Table 2). This decay coincides with a decline in the GSB (∼600 nm) and a rise of a new distinct peak at 617 nm (Figure 6a, EAS C). The latter is assigned to the excited state absorption of state C, and it remains essentially constant on the time scale of the fsTA experiment (up to 1.2 ns) (see Supporting Information 9). The nsTA spectra at 50 ns (Figure S20), the earliest time point in our nsTA system, nearly matches EAS C (Figure 6), indicating that the state probed by nsTA is the same as state C deduced from the fsTA experiments. The lifetime of state C was determined to be ∼4.2 μs in deaerated and ∼0.3 ns in aerated TolH solutions. Therefore, state C is most likely a triplet state, and its decay to the ground state is dominated by T_1_ → S_0_ intersystem crossing (*k*_isc_). No phosphorescence could be observed from Zn­(BDP)_2_ even at −196 °C (spectral range probed 700–1000 nm).

The decay of the intermediate state B to the triplet state C (*k*_T_, Figure 7) could be induced by metal-enhanced spin–orbit coupling as well as other processes facilitating spin mixing in radical pairs.^76^ The balance between the singlet (*k*_S_) and triplet (*k*_T_) charge recombination processes (Figure 7) would depend on the energies of the B and C states, and as such it could be influenced by the solvent. If state B were polar, in MeCN (ε = 37.5) and other polar solvents its energy could fall below that of the triplet C, so that the fast recombination to the ground state would become the dominant pathway, as it appears to be the case for Zn­(BDP)_2_. It is important to note that the rate-determining factor for the triplet formation (*k*_T_), which also accelerates with solvent polarity (Table 2), could be related to the spin dynamics of state B state rather than B → C transition itself. In this regard, the environment could have its own effect(s) on the spin mixing with more polar solvents favoring faster singlet–triplet interconversion and thus accelerating the decay.

### Origin of Fluorescence

A pertinent question in the context of fsTA data is the origin of fluorescence of Zn­(BDP)_2_. As noted in Table 1, the fluorescence rates increase with the solvent polarity, which at first glance suggests that fluorescence arises from a state with polar/CT features. However, the fluorescence spectra do not shift to the red (Figure 2), which would be expected if the emissive state is polar in nature. Two different scenarios could fit these observations. One is that state B is a nonpolar weakly fluorescent excimer-like state, whose energy per se is not affected by the solvent polarity, but its decay, occurring via a collapse to a CT state and subsequent nearly instant charge recombination (to either a triplet or ground state), is influenced by the solvent, with more polar solvents facilitating the collapse.

The second scenario is depicted in Figure 7. Here the fluorescence is emitted from the lower-lying Frenkel exciton state (*e*_ant_) that exists in “fast” equilibrium with state B, *k*_f_≫(*k*_S_ + *k*_T_), *k*_r_≫(*k*_S_ + *k*_T_), such that the equilibrium is established and maintained during the lifetime of state B. Here again state B is an excimer-like state that is sensitive to the solvent polarity. As the Frenkel exciton state is nonpolar, its energy is not correlated with the solvent polarity so the linear fluorescence spectra would not have a solvent dependent shift, however, its decay would be accelerated as state B becomes stabilized in polar solvents. This second scenario formally would be analogous to thermally activated delayed fluorescence (TADF),^77,78^ where the fluorescence from a singlet state (S_1_) reflects the slow decay of a triplet state (T_1_), while these two states exist in equilibrium due to fast forward and reverse intersystem crossing.

In the second scenario (Figure 7), the fluorescence decay from A (fl) would be expected to be biphasic, where the first prompt phase would parallel the initial rise of the longer-lived state, i.e. state B of Zn­(BDP)_2_ in the present case. However, the instrument response function of the TCSPC system employed in our experiments was on the order of ∼100 ps, which is much longer than the decay of the exciton state(s) (tens of picoseconds). As a result, the initial fast decay of the fluorescence signal could not be observed. We performed kinetic simulations according to the scheme shown in Figure 7, where the values of the constants *k*_f_ and *k*_isc_ were taken from the fsTA (τ_1_, Table 2) and nsTA measurements, respectively, while the other constants were manually adjusted, so that the simulated traces approximately reproduced the experimentally observed fsTA and fluorescence dynamics (Supporting Information 10). The resulting rate constants were all within the reasonable range, strengthening confidence in the feasibility of the proposed mechanism.

Overall, the scheme in Figure 7 echoes the relaxation pathways proposed for nonextended Zn bis-dipyrrinates and bis-BODIPY derivatives,^22,32,33^ albeit with one important distinction. In the case of Zn­(BDP)_2_, initial excitation produces not a state confined to a single chromophore, but a highly delocalized state formed due to exciton coupling and encompassing both ligands. The nature of the subsequent state B could vary from a zwitter ion/biradical, where the charges are fully separated and localized to the individual BDP units, to an excimer-like state, which is a superposition of the FE states and CTE states, with the respective contributions dependent on the environment. Such mixed states have been recently conceptualized in the context of photoinduced processes in dimeric systems.^79^

The transition from the initial exciton state to the intermediate state (state B) could be induced by fluctuations of the solvent and/or structural changes that occur within the molecule itself. In this regard, according to the TDDFT calculations, in the optimized excited state geometry of Zn­(BDP)_2_, the dihedral angle between the BDP ligands is consistently smaller than in the ground state. In the case of wB97XD/cc-pVTZ model chemistry, the angle decreases from 52.66° (S_0_) to 37.04° (S_1_) (Supporting Information 3). The contact between the frontier orbitals and the electronic coupling between the BDP ligands is likely to increase at smaller angles as the Zn­(BDP)_2_ system relaxes along the excited state potential energy surface, which could lead to the formation of the intermediate state B with FE and CTE features.

The structural origin of exciton coupling in Zn­(BDP)_2_ is the nonplanar orientation of the BDP ligands. However, the root cause of this nonplanarity is not well understood. Since a number of nonextended Zn bis-dipyrrinates have been reported to be orthogonal, and no apparent signs of exciton coupling have been detected in these molecules, it is tempting to assume that there exists a link between π-extension (benzo-extension in the case of BDP) and the propensity of the ligands to align in plane with one-another, which underpins distortion of Zn­(BDP)_2_ from the ideal orthogonal geometry to the oblique geometry that facilitates excitonic coupling. However, a recently reported non-π-extended dipyrrin,^30^ in which the pyrrolic units are fused with nonaromatic cyclohexeno-rings, shows even stronger deviation from orthogonal geometry than Zn­(BDP)_2_ (dihedral angle θ = 55° vs 65°, based on X-ray crystallographic data^29,30^ ) and, similarly, strong signs of excitonic coupling. Common features of the two structures are alkoxycarbonyl groups in 2,2′-positions and the presence of *meso*-aryl groups. However, it is not immediately clear how these substituents can influence the geometry of bis-complexes. Further structural and computational studies will be required to delineate the effects of substituents on the geometry of these types of dipyrrinates.

## Conclusions

In this study, we used an array of spectroscopic methods alongside theoretical modeling and computations to investigate excitation dynamics in Zn complexes of a π-extended dipyrrin, Zn­(BDP)­X and Zn­(BDP)_2_. Notably, the initial excited states in these complexes were characterized using 2DES, which, to the best of our knowledge, presents the first example of the application of 2DES to metal complexes of dipyrrins and, more broadly, to investigation of excitonic coupling among ligands in metal complexes where the ligands are held together by a single photoinert metal ion. The photophysical behavior of Zn­(BDP)_2_ was found to be consistent with predictions of the molecular exciton model,^34,45^ including a substantial decrease in fluorescence. The excitation dynamics, followed by means of fsTA and nsTA, revealed that the relaxation pathway of Zn­(BDP)_2_ includes a short-lived intermediate state, likely possessing CTE character and possibly resembling SBCT states proposed earlier for orthogonal bis-dipyrrinates.^22,33^ This intermediate state further reduces emissivity of Zn­(BDP)_2_ by promoting, in a solvent-dependent manner, relaxation to the ground state and/or intersystem crossing to the triplet state. In addition to bringing new valuable information on the photophysics of dipyrrins, our study demonstrates that metallodipyrrinates present a versatile synthetically tunable model for investigation of exciton relaxation, charge transfer, and triplet formation i.e. the primary processes in solar energy conversion, artificial photosynthetic systems, photovoltaics, and photocatalysts.

## Supplementary Material



## Notes

Pulse characterizations, fluorescence emission and excitation spectra, computed geometries and TDDFT spectra, simulated electronic absorption spectrum of Zn­(BDP)_2_, *t*_2_ dependent 2DES, fsTA spectra of Zn­(BDP)_2_, global analysis results, electrochemical data, nsTA spectra for Zn­(BDP)_2_, kinetic simulations, time-resolved fluorescence measurements (PDF)

## ¶

Institute of Pharmacology Please check: && Toxicology, University of Zurich, Winterthurerstrasse 190, 8057 Zürich, Switzerland
