# Supplementary material for: Exciton Coupling and Charge Transfer Dynamics in Zn(II) Complexes of π‑Extended Dipyrrins
Source: J Phys Chem B. 2025 Jun 13;129(39):10049–62. doi: 10.1021/acs.jpcb.5c03312 (PMC12498501; doi:10.1021/acs.jpcb.5c03312)
Supplement: Supplementary file 1 [file jp5c03312_si_001.pdf]

Supporting Information

Exciton Coupling and Charge Transfer Dynamics in Zn(II) Complexes of π-Extended Dipyrrins

Dabin Kim,[1§] Luca Ravotto,[1,2§] April Bialas,[3] Thomas Troxler,[1,2] Zhongping Ou,[4] Karl M. Kadish,[4] Andrei V. Cheprakov,[5] Frank C. Spano,[3] Sergei A. Vinogradov,[1,2] Jessica M. Anna[1,6*]*

[1] Department of Chemistry, School of Arts and Sciences, University of Pennsylvania, Philadelphia, PA 19104, United States

[2] Department of Biochemistry and Biophysics, Perelman School of Medicine, University of Pennsylvania, Philadelphia, PA 19104, United States

[3] Department of Chemistry, Temple University, Philadelphia, PA 19122, United States

[4] Department of Chemistry, University of Houston, Houston, TX 77204, United States

[5] Department of Chemistry, Moscow State University, 119899 Moscow, Russia.

[6] Department of Chemistry, University of Pittsburgh, Pittsburgh, PA 15216, United States

§ equal contributions.

* corresponding authors.

Table of contents

SI 1. Pulse Characterization	S3
SI 2. Fluorescence Excitation Spectra	S4
SI 3. Optimized Geometries and TDDFT Spectra	S5
SI 4. Frenkel-Holstein Hamiltonian Simulations	S17
SI 5. Waiting Time (t_2) Dependent 2DES Spectra of Zn(BDP)Cl & $Zn(BDP)_2$	S21
SI 6. fsTA Spectra of $Zn(BDP)_2$ in Et_2O and cHex	S23
SI 7. Decay Associated Spectra (DAS) and Evolution Associated Spectra (EAS)	S24
SI 8. Electrochemical Data	S25
SI 9. nsTA Spectra of $Zn(BDP)_2$	S26
SI 10. Kinetic Simulations	S29
SI 11. Time-Resolved Fluorescence Measurements of $Zn(BDP)_2$	S30

SI 1. Pulse Characterization

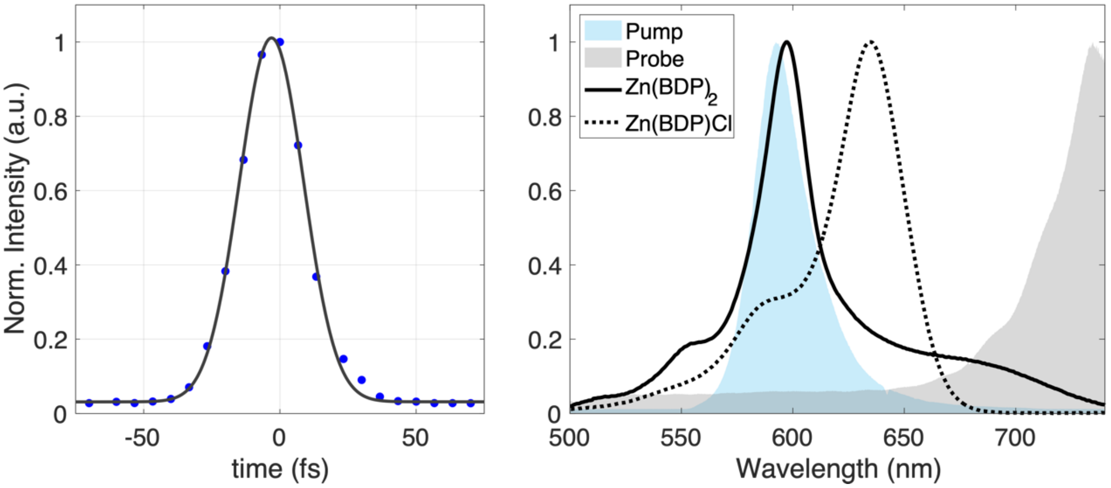

Figure S1. (left) Projection of SHG-FROG onto the time axis for the NOPA pump pulse used for the fsTA. (right) The NOPA pump pulse (blue) and the white-light probe pulse (grey) used to perform fsTA measurements on $Zn(BDP)_2$ and Zn(BDP)Cl.

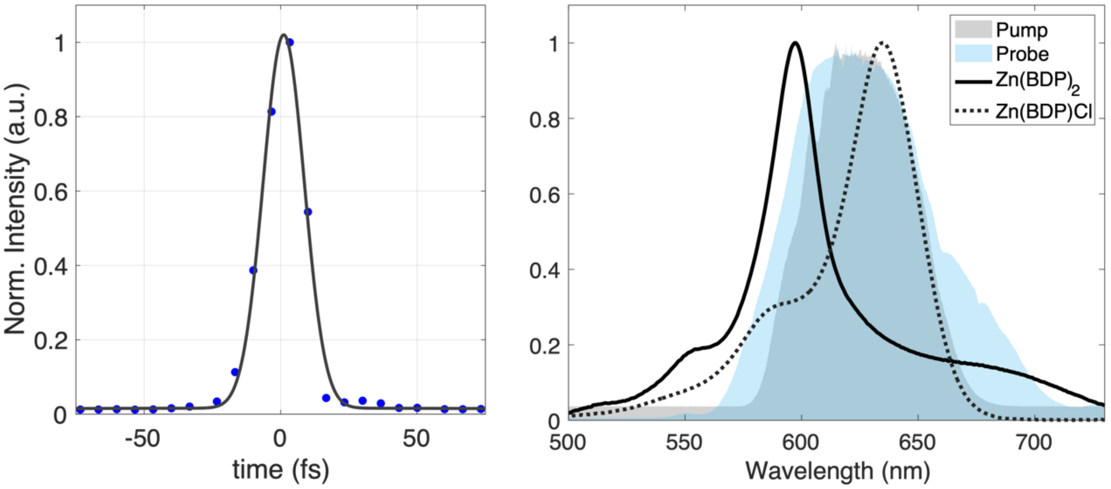

Figure S2. (left) Projection of SFG-FROG onto the time axis for the Dazzler pump and NOPA probe pulses used for the 2DES measurements. (right) The Dazzler pump pulse (grey) and the NOPA probe pulse (blue) used to perform the 2DES measurements on $Zn(BDP)_2$ and Zn(BDP)Cl.

SI 2. Fluorescence Excitation Spectra

The fluorescence excitation spectra of Zn(BDP)Cl in pyridine and $Zn(BDP)_2$ in diethyl ether, toluene and cyclohexane are shown in Fig. S3. The excitation spectra resemble the absorption spectra, indicating that the photons absorbed by the different transitions, which give rise to the peaks in the absorption spectra, contribute to the fluorescence spectra. No emission at 770 nm could be detected from $Zn(BDP)_2$ in acetonitrile.

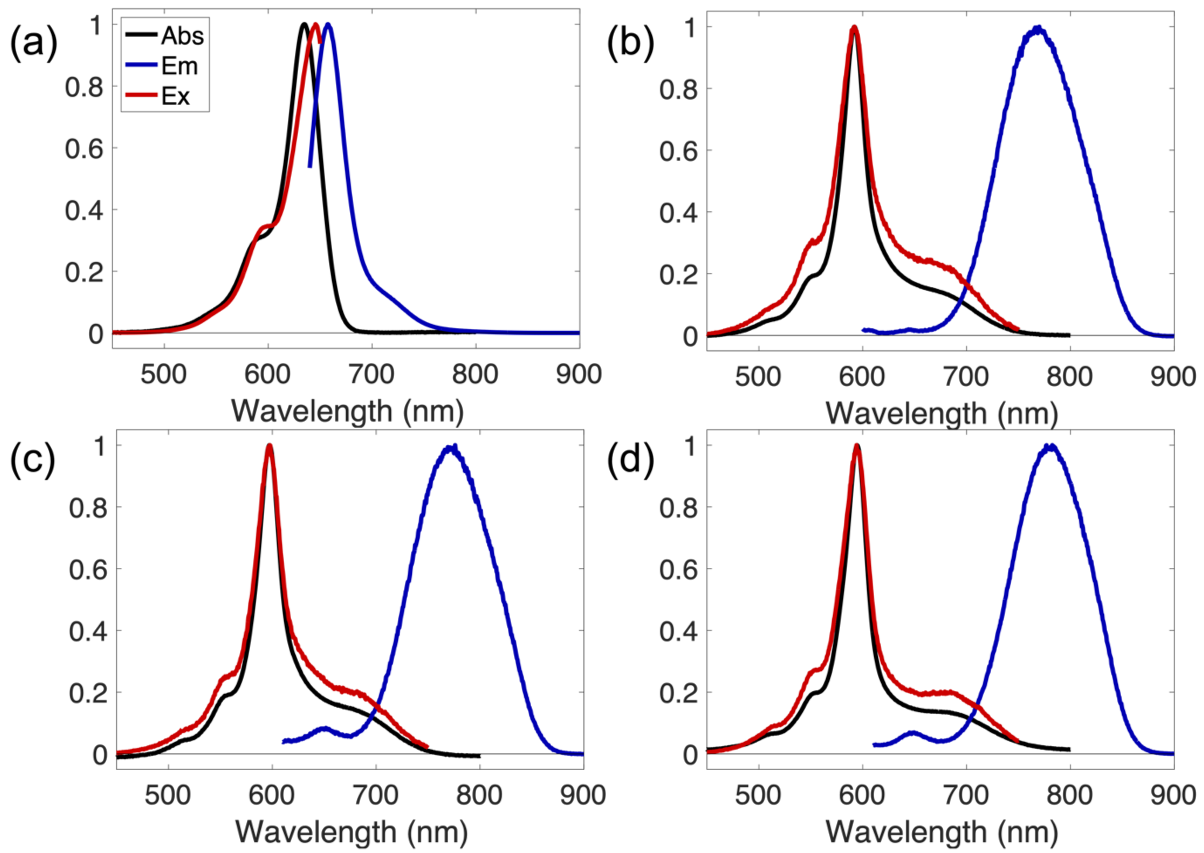

Figure S3. The absorption (black), emission (blue) and excitation (red) spectra of Zn(BDP)Cl in pyridine (a) and $Zn(BDP)_2$ in diethyl ether (Et_2O, (b)), toluene (TolH,(c)), and cyclohexane (cHex,(d)).

SI 3. Optimized Geometries and TDDFT Spectra

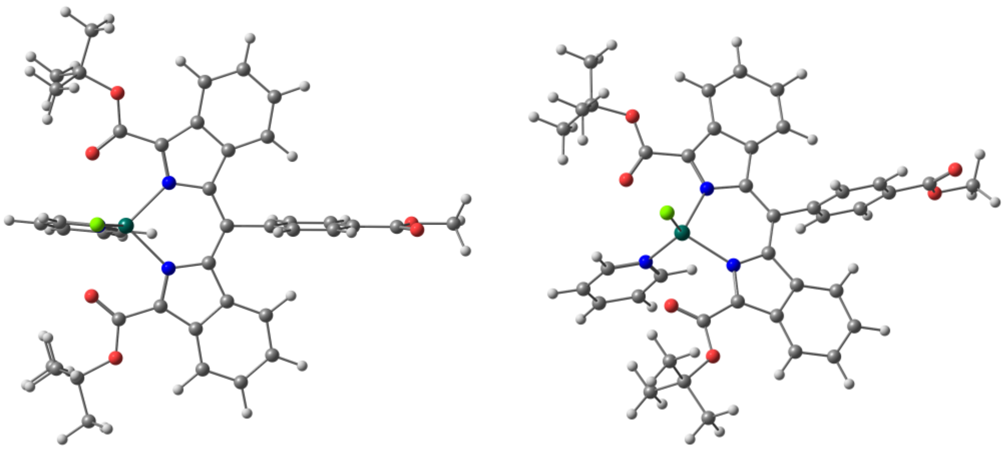

Figure S4. Optimized geometry of Zn(BDP)Cl·Pyr in the ground electronic state (wB97XD/cc-pVTZ) in TolH (modeled as PCM).

Table S1. Atomic coordinates Zn(BDP)Cl·Pyr (wB97XD/cc-pVTZ) in TolH (modeled as PCM).

#	Atomic number	x (Å)	y (Å)	z (Å)
1	30	1.82645	0.06108	-0.49412
2	8	-7.8584	-0.11095	1.19082
3	8	-8.0174	-0.17849	-1.03916
4	8	3.02021	-2.33074	-0.36468
5	8	2.0495	4.56784	-0.3037
6	8	2.92131	2.50782	-0.02847
7	8	2.23468	-4.44243	-0.42895
8	7	0.34628	1.47879	-0.1973
9	7	0.40277	-1.42328	-0.29885
10	6	4.3582	-4.78325	0.73933
11	6	3.17336	-6.57164	-0.55041
12	6	4.25372	-4.68224	-1.78926
13	6	4.08829	5.09851	0.94665
14	6	2.895	6.7273	-0.53195
15	6	4.12674	4.80882	-1.56911
16	6	-9.28283	-0.13939	1.27329
17	6	-7.34433	-0.13501	-0.0405
18	6	-5.85481	-0.10225	-0.03728
19	6	-5.11793	-0.05023	1.14171
20	6	-5.19658	-0.12471	-1.26222
21	6	-3.73626	-0.01971	1.09146
22	6	-3.81555	-0.09686	-1.3099
23	6	-3.0808	-0.04336	-0.13242
24	6	2.10808	-3.12521	-0.37062
25	6	3.54261	-5.09635	-0.5084
26	6	3.32882	5.27919	-0.36096
27	6	1.97915	3.25352	-0.16219

28	6	-3.25835	4.35498	-0.13208
29	6	-2.22142	5.29912	-0.11884
30	6	-0.91012	4.89202	-0.13177
31	6	-3.00536	3.00247	-0.15306
32	6	-2.87275	-3.08437	-0.06626
33	6	-3.06802	-4.44535	-0.03226
34	6	-1.99397	-5.34683	-0.08834
35	6	-0.70425	-4.88674	-0.17945
36	6	-1.67369	2.55795	-0.16256
37	6	-0.6391	3.519	-0.15689
38	6	0.57951	2.77549	-0.17717
39	6	-1.01608	1.2622	-0.18671
40	6	-1.59408	-0.01141	-0.18409
41	6	-0.49058	-3.50291	-0.21522
42	6	-1.56314	-2.58425	-0.16056
43	6	-0.96198	-1.26407	-0.21541
44	6	0.69235	-2.71159	-0.3001
45	1	4.65283	-3.73812	0.77054
46	1	5.25688	-5.40013	0.73882
47	1	3.78344	-5.01949	1.63522
48	1	2.63104	-6.8572	0.35085
49	1	4.07677	-7.17659	-0.6174
50	1	2.54848	-6.78441	-1.41725
51	1	4.52792	-3.63126	-1.77281
52	1	3.61421	-4.86678	-2.65219
53	1	5.15872	-5.27966	-1.90074
54	1	4.95395	5.76098	0.94646
55	1	4.43234	4.07559	1.06986
56	1	3.45465	5.36654	1.79239
57	1	3.77223	7.36954	-0.59962
58	1	2.29384	7.05082	0.31776
59	1	2.30737	6.84496	-1.44182
60	1	3.52947	4.90204	-2.47575
61	1	4.44237	3.77484	-1.4627
62	1	5.01117	5.43716	-1.67549
63	1	-9.51725	-0.11339	2.33242
64	1	-9.67272	-1.04869	0.81975
65	1	-9.71117	0.72396	0.76766
66	1	-5.62676	-0.03247	2.09361
67	1	-5.7801	-0.16472	-2.17061
68	1	-3.15996	0.02361	2.00518
69	1	-3.30107	-0.11609	-2.26043
70	1	-2.4599	6.35377	-0.10336
71	1	-0.10218	5.60682	-0.12969
72	1	-4.28479	4.69661	-0.12836
73	1	-3.83567	2.31703	-0.16677
74	1	-2.18808	-6.41036	-0.0615
75	1	0.13135	-5.56745	-0.22614
76	1	-3.72992	-2.4343	-0.02263
77	1	-4.07714	-4.82896	0.03731
78	17	2.81676	0.24572	-2.47626
79	6	4.05337	0.08094	1.51235
80	6	4.68138	-0.03781	2.73671
81	6	3.91203	-0.30148	3.85792
82	6	2.54167	-0.43738	3.71404
83	6	1.99387	-0.30684	2.45227

84	7	2.73462	-0.05272	1.37503
85	1	4.60779	0.28878	0.60825
86	1	5.75304	0.07498	2.80343
87	1	4.37418	-0.3997	4.83037
88	1	1.90313	-0.64243	4.5601
89	1	0.92911	-0.41091	2.2876

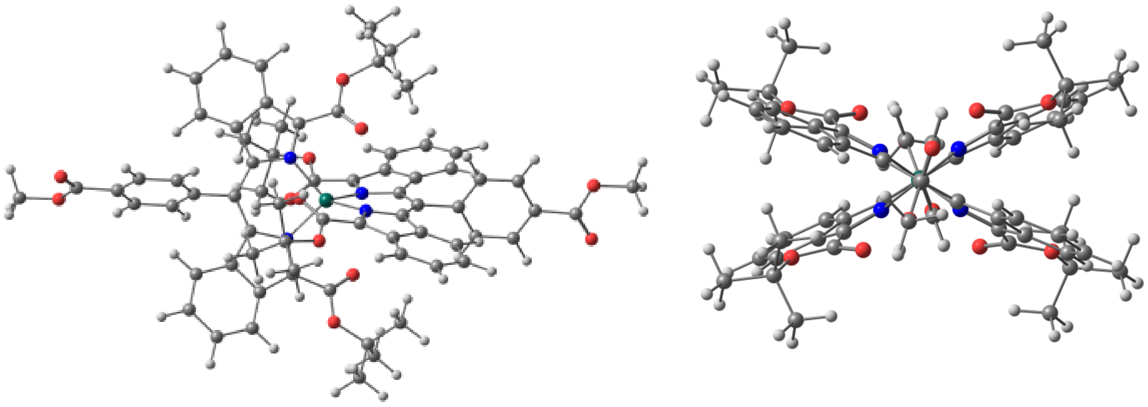

Figure S5. Optimized geometry of $Zn(BDP)_2$ in the ground electronic state (wB97XD/cc-pVTZ) in TolH (modeled as PCM).

Table S2. Atomic coordinates of $Zn(BDP)_2$ (wB97XD/cc-pVTZ) in TolH (modeled as PCM).

#	Atomic number	x (Å)	y (Å)	z (Å)
1	30	6E-6	0.01038	4.16E-4
2	8	9.64984	-0.36568	-0.88431
3	8	9.68839	0.37651	1.22515
4	8	-9.64984	-0.36612	0.88385
5	8	-9.68829	0.37609	-1.2256
6	8	0.48745	-3.87554	-2.2095
7	8	1.17024	-1.73344	-2.01194
8	8	0.39519	3.89152	2.23831
9	8	1.0813	1.74857	2.06334
10	8	-0.39553	3.89134	-2.23797
11	8	-1.08156	1.74834	-2.06339
12	8	-0.48718	-3.87548	2.21004
13	8	-1.16992	-1.7334	2.01217
14	7	-1.35034	-1.16718	-0.91512
15	7	-1.38812	1.18945	0.85607
16	7	1.38798	1.18928	-0.85601
17	7	1.35056	-1.16714	0.91545
18	6	-2.11834	-3.7905	4.02724
19	6	-2.8514	-4.19021	1.64004
20	6	-1.4647	-5.8953	2.83643
21	6	-1.34876	5.90634	-2.91639
22	6	-2.78223	4.20447	-1.7716
23	6	-1.94738	3.79463	-4.12341

24	6	2.78178	4.20487	1.7714
25	6	1.34846	5.90649	2.91677
26	6	1.94751	3.79464	4.12333
27	6	2.1181	-3.79092	-4.02719
28	6	2.85184	-4.19018	-1.64014
29	6	1.46477	-5.89548	-2.83581
30	6	11.07664	-0.37454	-0.88594
31	6	9.06959	0.03663	0.24816
32	6	-11.07664	-0.37508	0.88538
33	6	-9.06953	0.03623	-0.24857
34	6	1.76531	-4.4121	-2.68255
35	6	-3.76477	-4.80877	-2.34065
36	6	-4.82335	-4.05029	-1.81738
37	6	-2.48097	-4.32284	-2.31206
38	6	0.31965	-2.58755	-1.93768
39	6	-4.61946	-2.80418	-1.27144
40	6	-2.25884	-3.06195	-1.74682
41	6	-1.07311	-2.30048	-1.52739
42	6	-3.31726	-2.28157	-1.23109
43	6	-4.89216	4.08176	1.59358
44	6	1.65281	4.42344	2.7683
45	6	-3.85733	4.838	2.16529
46	6	-2.70669	-1.07673	-0.70315
47	6	-4.66621	2.83418	1.05991
48	6	-2.57489	4.34866	2.19776
49	6	-3.32163	0.01301	-0.0737
50	6	-3.36499	2.30806	1.08174
51	6	0.23693	2.60514	1.95423
52	6	-2.3296	3.08639	1.64517
53	6	-2.73367	1.10166	0.58283
54	6	-1.13666	2.32214	1.48062
55	6	-1.65304	4.42325	-2.76821
56	6	3.85687	4.83797	-2.16553
57	6	4.89178	4.08185	-1.59384
58	6	2.57447	4.34852	-2.19791
59	6	-0.23722	2.60491	-1.95414
60	6	4.66596	2.83426	-1.06011
61	6	2.3293	3.08627	-1.64523
62	6	1.13642	2.32191	-1.48057
63	6	3.36478	2.30804	-1.08184
64	6	4.82369	-4.05027	1.8171
65	6	-1.76517	-4.41194	2.68282
66	6	3.76517	-4.80882	2.34041
67	6	2.73359	1.1016	-0.58284
68	6	4.61971	-2.80413	1.27126
69	6	2.48136	-4.32294	2.31197
70	6	3.32168	0.01308	0.07369
71	6	3.31748	-2.28156	1.23104
72	6	-0.31934	-2.58754	1.93798
73	6	2.25912	-3.06201	1.74682
74	6	2.70684	-1.07668	0.7033
75	6	1.07338	-2.30052	1.52764
76	6	4.80755	0.0143	0.10669
77	6	5.47795	0.39271	1.26319
78	6	5.52757	-0.36272	-1.01942
79	6	6.85949	0.40012	1.29019

80	6	6.91017	-0.36829	-0.99038
81	6	7.58248	0.01691	0.16538
82	6	-4.8075	0.01416	-0.10681
83	6	-5.47784	0.39261	-1.26333
84	6	-5.52757	-0.36297	1.01923
85	6	-6.85939	0.39994	-1.29042
86	6	-6.91017	-0.36861	0.9901
87	6	-7.58243	0.01661	-0.16569
88	1	-2.99082	-4.30039	4.43607
89	1	-2.34837	-2.73329	3.92673
90	1	-1.29296	-3.91014	4.7293
91	1	-2.53676	-4.57158	0.6686
92	1	-3.09596	-3.13727	1.53605
93	1	-3.74889	-4.72998	1.94282
94	1	-2.35502	-6.41826	3.18359
95	1	-0.66615	-6.05308	3.5613
96	1	-1.16064	-6.3228	1.8815
97	1	-0.52028	6.06209	-3.60736
98	1	-1.08659	6.33936	-1.95158
99	1	-2.22445	6.42561	-3.30397
100	1	-3.02688	3.15128	-1.67088
101	1	-3.66803	4.73802	-2.11672
102	1	-2.51125	4.59411	-0.79031
103	1	-1.0925	3.91157	-4.78972
104	1	-2.80167	4.30181	-4.57202
105	1	-2.18103	2.73776	-4.02798
106	1	3.0265	3.15172	1.67054
107	1	3.66762	4.73843	2.11639
108	1	2.51057	4.59459	0.79019
109	1	0.52014	6.06208	3.60796
110	1	1.08601	6.33964	1.95208
111	1	2.22421	6.42576	3.30421
112	1	1.09277	3.91141	4.78986
113	1	2.80187	4.30179	4.57181
114	1	2.18119	2.73779	4.02766
115	1	2.99033	-4.30106	-4.43626
116	1	2.34838	-2.73374	-3.92694
117	1	1.29245	-3.9105	-4.72893
118	1	2.53754	-4.57142	-0.66854
119	1	3.09633	-3.13719	-1.53639
120	1	3.74929	-4.72989	-1.94313
121	1	2.35503	-6.41854	-3.18295
122	1	0.66614	-6.0534	-3.56056
123	1	1.16081	-6.32276	-1.88075
124	1	11.36818	-0.71755	-1.87324
125	1	11.45442	-1.05106	-0.12155
126	1	11.46423	0.62547	-0.70086
127	1	-11.36823	-0.71807	1.87267
128	1	-11.45431	-1.05166	0.12099
129	1	-11.46429	0.62489	0.70024
130	1	-3.96495	-5.78373	-2.7636
131	1	-5.82639	-4.45477	-1.84302
132	1	-1.65402	-4.89914	-2.69758
133	1	-5.46255	-2.26144	-0.87764
134	1	-5.89385	4.4897	1.56968
135	1	-4.07431	5.8144	2.57646

136	1	-5.49102	2.29408	0.62592
137	1	-1.7659	4.92399	2.62078
138	1	4.07374	5.81438	-2.57675
139	1	5.89344	4.48985	-1.57002
140	1	1.76541	4.92378	-2.62091
141	1	5.49084	2.29423	-0.62617
142	1	5.82675	-4.45471	1.84265
143	1	3.96543	-5.78379	2.76328
144	1	5.46274	-2.26133	0.87741
145	1	1.65445	-4.89929	2.69751
146	1	4.91062	0.68001	2.13757
147	1	7.3943	0.69737	2.18067
148	1	-4.91047	0.68001	-2.13766
149	1	-7.39415	0.69721	-2.18092
150	1	4.99817	-0.65099	-1.91699
151	1	7.46981	-0.66623	-1.8641
152	1	-7.46986	-0.66663	1.86376
153	1	-4.99822	-0.65125	1.91682

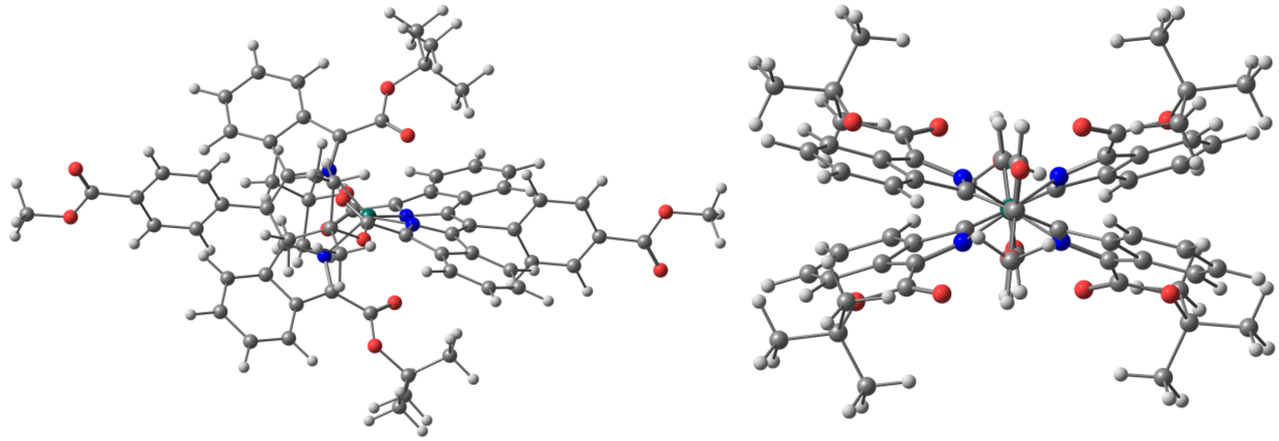

Figure S6. Optimized geometry of $Zn(BDP)_2$ in the S_1 excited state (wB97XD/cc-pVTZ) in TolH (modeled as PCM).

Table S3. Atomic coordinates of $Zn(BDP)_2$ in S_1 state (wB97XD/cc-pVTZ) in TolH (modeled as PCM).

#	Atomic number	x (Å)	y (Å)	z (Å)
1	30	-6.77E-4	-0.00679	-3.01E-4
2	8	9.63821	-0.11825	-0.93395
3	8	9.6738	0.14904	1.28673
4	8	-9.64052	-0.13115	0.92296
5	8	-9.67385	0.13972	-1.29732
6	8	0.46601	-3.94525	-2.23735
7	8	1.12234	-1.78773	-2.05298
8	8	0.3795	3.95028	2.19654
9	8	1.03686	1.78877	2.07234

10	8	-0.38779	3.95327	-2.19065
11	8	-1.04218	1.79069	-2.06926
12	8	-0.45614	-3.94669	2.24209
13	8	-1.11714	-1.79059	2.05761
14	7	-1.33513	-1.22614	-0.84382
15	7	-1.37197	1.21546	0.7735
16	7	1.36841	1.21862	-0.77313
17	7	1.33616	-1.22546	0.84071
18	6	-1.67586	-3.71225	4.34225
19	6	-2.89797	-4.2028	2.18438
20	6	-1.33723	-5.89421	3.16026
21	6	-1.2541	5.91732	-3.08714
22	6	-2.83161	4.2034	-2.17984
23	6	-1.56134	3.75859	-4.31986
24	6	2.82297	4.20389	2.1879
25	6	1.24239	5.91406	3.09691
26	6	1.55176	3.75372	4.32625
27	6	1.70129	-3.71284	-4.32868
28	6	2.90758	-4.19771	-2.16052
29	6	1.35678	-5.89315	-3.14532
30	6	11.0648	-0.11444	-0.93737
31	6	9.05615	0.02461	0.25896
32	6	-11.06712	-0.12881	0.92476
33	6	-9.05724	0.01423	-0.26905
34	6	1.63837	-4.40758	-2.97393
35	6	-3.69712	-5.08819	-1.65667
36	6	-4.74485	-4.30489	-1.16983
37	6	-2.42191	-4.57122	-1.76917
38	6	0.29903	-2.66559	-1.89929
39	6	-4.54333	-2.98844	-0.79526
40	6	-2.20605	-3.25009	-1.38032
41	6	-1.03667	-2.42175	-1.34694
42	6	-3.26444	-2.44668	-0.90435
43	6	-4.78529	4.30432	0.91502
44	6	1.52973	4.42607	2.9592
45	6	-3.75897	5.08988	1.44233
46	6	-2.66929	-1.1572	-0.56796
47	6	-4.57017	2.98417	0.56154
48	6	-2.49156	4.57126	1.61782
49	6	-3.31091	-0.00472	-0.08083
50	6	-3.29904	2.44049	0.73445
51	6	0.21967	2.6645	1.87943
52	6	-2.26168	3.24596	1.25151
53	6	-2.69134	1.14796	0.43393
54	6	-1.09368	2.41547	1.27738
55	6	-1.53924	4.42869	-2.95169
56	6	3.74971	5.09728	-1.43766
57	6	4.77749	4.31227	-0.9124
58	6	2.48286	4.57725	-1.61302
59	6	-0.226	2.6672	-1.87566
60	6	4.5644	2.99126	-0.56087
61	6	2.25503	3.25106	-1.24865
62	6	1.08814	2.41902	-1.27498
63	6	3.29387	2.44616	-0.73365
64	6	4.74989	-4.30084	1.15679
65	6	-1.62227	-4.40936	2.98832

66	6	3.70394	-5.08582	1.64472
67	6	2.68811	1.15236	-0.43473
68	6	4.54626	-2.98412	0.7843
69	6	2.42842	-4.57032	1.76037
70	6	3.30962	-4.72E-4	0.07733
71	6	3.26701	-2.44386	0.89653
72	6	-0.2937	-2.66759	1.89973
73	6	2.21038	-3.24898	1.37345
74	6	2.66991	-1.15449	0.56326
75	6	1.04003	-2.42188	1.34337
76	6	4.78981	0.00343	0.11161
77	6	5.46293	0.13397	1.32183
78	6	5.51692	-0.12265	-1.06681
79	6	6.84387	0.14256	1.35239
80	6	6.89902	-0.12264	-1.03893
81	6	7.56982	0.01236	0.17286
82	6	-4.79106	-0.00247	-0.1168
83	6	-5.46293	0.12929	-1.32759
84	6	-5.51939	-0.13129	1.06056
85	6	-6.84384	0.1364	-1.35973
86	6	-6.90145	-0.13277	1.03109
87	6	-7.57101	0.00347	-0.18125
88	1	-2.46411	-4.16262	4.94574
89	1	-1.88502	-2.65152	4.23141
90	1	-0.72928	-3.8347	4.8691
91	1	-2.80188	-4.64243	1.1916
92	1	-3.13137	-3.14838	2.07517
93	1	-3.72345	-4.69626	2.69818
94	1	-2.15007	-6.36603	3.71123
95	1	-0.41038	-6.04653	3.71346
96	1	-1.24917	-6.3789	2.18816
97	1	-0.31631	6.08287	-3.6177
98	1	-1.18744	6.38201	-2.10364
99	1	-2.05644	6.39885	-3.64504
100	1	-3.06617	3.14666	-2.09866
101	1	-3.6466	4.70722	-2.70025
102	1	-2.75701	4.62147	-1.17593
103	1	-0.60351	3.89283	-4.8231
104	1	-2.33713	4.21913	-4.93172
105	1	-1.77099	2.69564	-4.2342
106	1	3.05906	3.14762	2.10512
107	1	3.63687	4.70798	2.70974
108	1	2.74849	4.62353	1.18464
109	1	0.30396	6.07741	3.62703
110	1	1.17583	6.38031	2.11413
111	1	2.04363	6.39578	3.65623
112	1	0.59339	3.88578	4.82902
113	1	2.32647	4.21432	4.93944
114	1	1.76297	2.6912	4.23898
115	1	2.49477	-4.16315	-4.92532
116	1	1.90808	-2.65161	-4.21817
117	1	0.75894	-3.83761	-4.86251
118	1	2.80442	-4.63559	-1.16766
119	1	3.13872	-3.14277	-2.05142
120	1	3.73768	-4.691	-2.66698
121	1	2.17447	-6.3648	-3.68921

122	1	0.43439	-6.04782	-3.70527
123	1	1.26201	-6.37619	-2.17303
124	1	11.35738	-0.23583	-1.97521
125	1	11.44973	-0.9354	-0.33528
126	1	11.44485	0.82607	-0.5428
127	1	-11.36076	-0.25216	1.96207
128	1	-11.45051	-0.94921	0.32092
129	1	-11.44769	0.81193	0.53125
130	1	-3.88502	-6.11361	-1.94492
131	1	-5.73449	-4.7316	-1.08204
132	1	-1.6056	-5.17499	-2.13258
133	1	-5.37478	-2.41601	-0.4183
134	1	-5.76838	4.73278	0.7765
135	1	-3.95714	6.11852	1.7116
136	1	-5.3846	2.41083	0.15027
137	1	-1.69066	5.17637	2.01206
138	1	3.9463	6.12662	-1.70545
139	1	5.76011	4.74182	-0.77394
140	1	1.68085	5.18191	-2.00568
141	1	5.37991	2.41837	-0.15113
142	1	5.73981	-4.72638	1.06657
143	1	3.89348	-6.11139	1.93136
144	1	5.3764	-2.41029	0.40657
145	1	1.61345	-5.17539	2.12465
146	1	4.89533	0.23076	2.23723
147	1	7.37567	0.24806	2.287
148	1	-4.89438	0.22822	-2.24217
149	1	-7.37468	0.24284	-2.29478
150	1	4.99073	-0.2226	-2.00635
151	1	7.45946	-0.22491	-1.95586
152	1	-7.46284	-0.23716	1.9472
153	1	-4.99417	-0.23219	2.00055

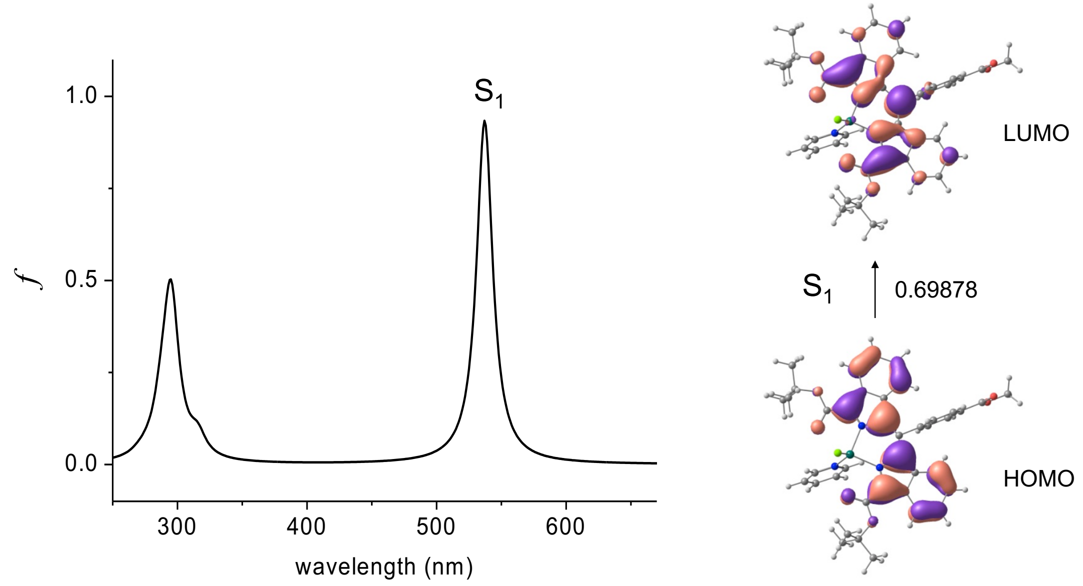

Figure S7. TDDFT spectrum of Zn(BDP)Cl·Pyr (wB97XD/cc-pVTZ) in TolH (modeled as PCM). The transitions are broadened by Lorentzians to facilitate visual comparison with the experimental absorption spectra. The S_1 transition is dominated by the single-electron HOMO $\rightarrow$LUMO excitation with the corresponding coefficient of 0.69878.

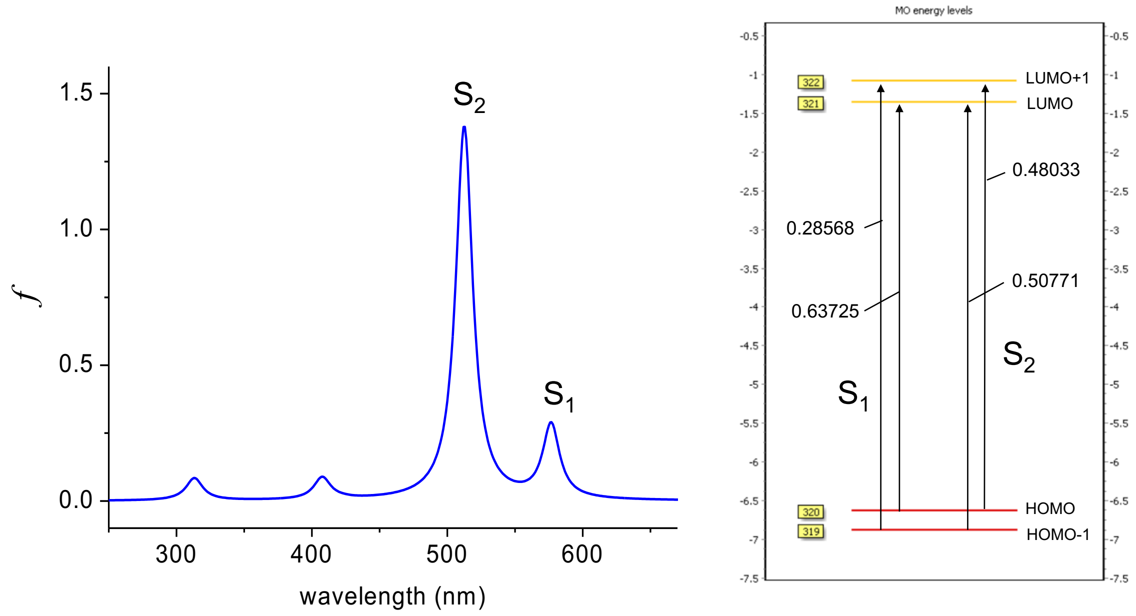

Figure S8. TDDFT spectrum of $Zn(BDP)_2$ (wB97XD/cc-pVTZ) in TolH (modeled as PCM). The transitions are broadened by Lorentzians to facilitate visual comparison with the experimental absorption spectra. The orbital diagram shows single-electron excitations (with the respective coefficients) that give rise to S_1 and S_2 Frenkel excitonic states. The corresponding orbitals (HOMO, HOMO-1, LUMO, LUMO+1) are shown in the figures below.

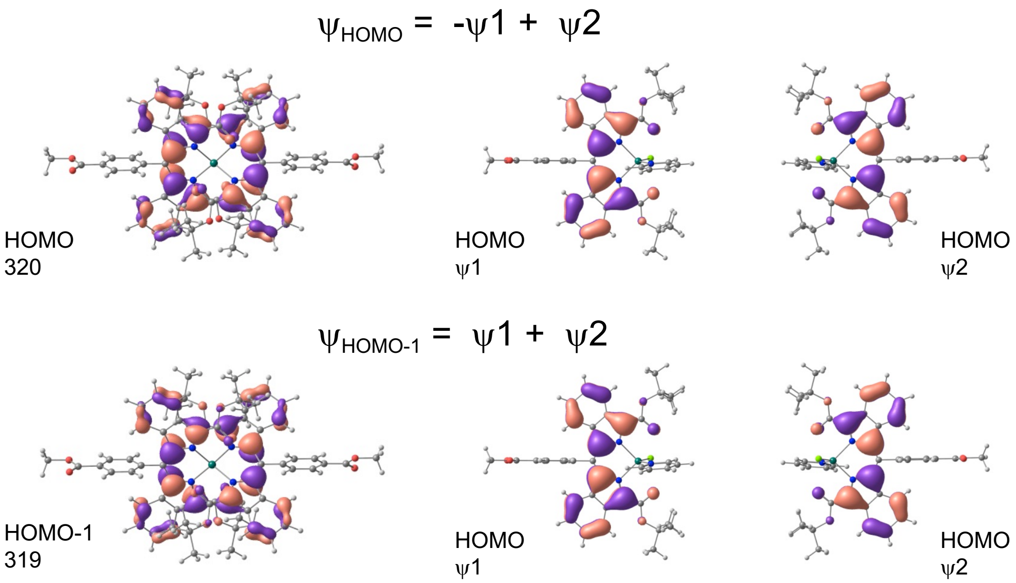

Figure S9. HOMO and HOMO-1 Kohn-Sham orbitals of $Zn(BDP)_2$ (left) and their "decomposition" into the orbitals of individual BDP ligands (right) computed for Zn(BDP)Cl·Pyr (wB97XD/cc-pVTZ).

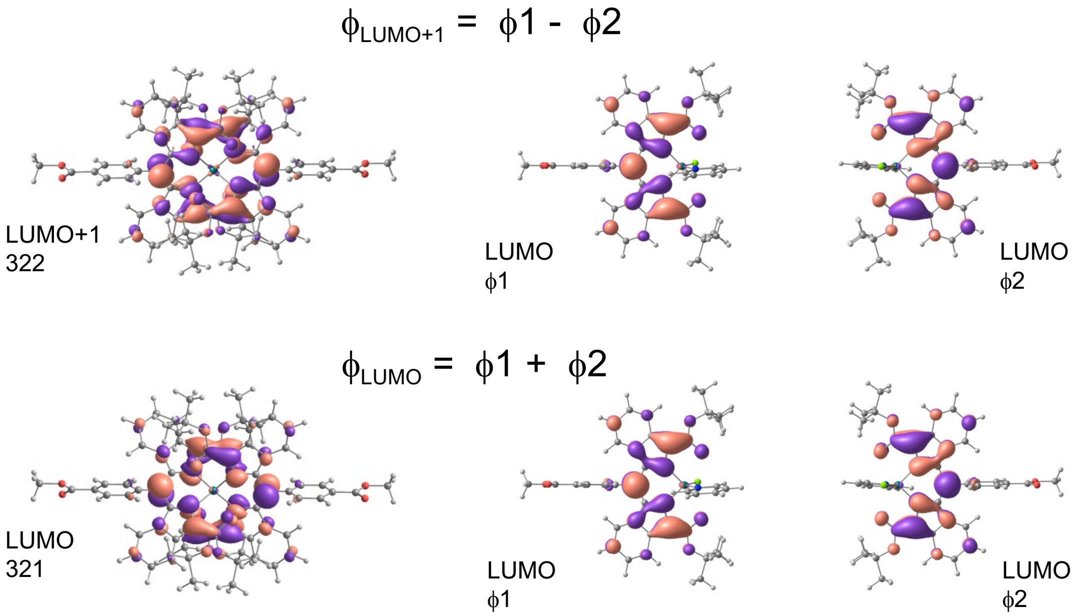

Figure S10. LUMO and LUMO+1 Kohn-Sham orbitals of $Zn(BDP)_2$ (left) and their "decomposition" into the orbitals of individual BDP ligands (right) computed for Zn(BDP)Cl·Pyr (wB97XD/cc-pVTZ).

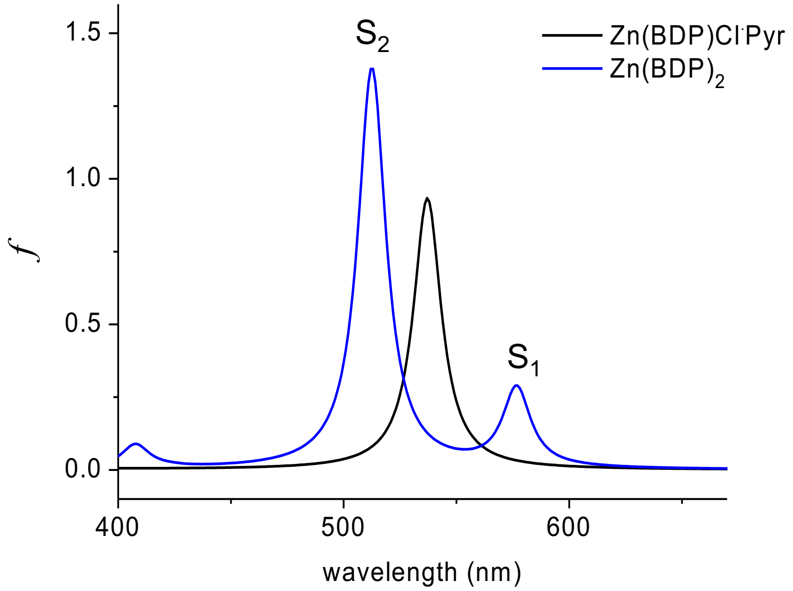

Figure S11. Superimposed TDDFT spectra of Zn(BDP)Cl·Pyr and $Zn(BDP)_2$ (wB97XD/cc-pVTZ) in TolH (modeled as PCM). The transitions are broadened by Lorentzians to facilitate visual comparison with the experimental absorption spectra.

SI 4. Frenkel-Holstein Hamiltonian Simulations

The simulated absorption spectrum of $Zn(BDP)_2$ in TolH (Fig. S12) was obtained using the parameters extracted from the DFT/TDDFT calculations, r=5.97Å, θ=52.66° (J_{12}=685 cm^{-1}), |μ|=10.32 Debye, HR factor=0.28, ω_{vib}=1250 cm^{-1}. The optical dielectric constant was ε_{op}=2.23 (ε_{op} value for toluene at 650 nm). The basis was constructed considering 6 vibrational states for each excited and ground electronic state of each ligand. The 12 lowest energy states, selected from the total of 42 states obtained upon diagonalization of the Hamiltonian, are given in Table S4. Four transitions were found to largely define the spectrum: 677, 640, 604, and 562 nm.

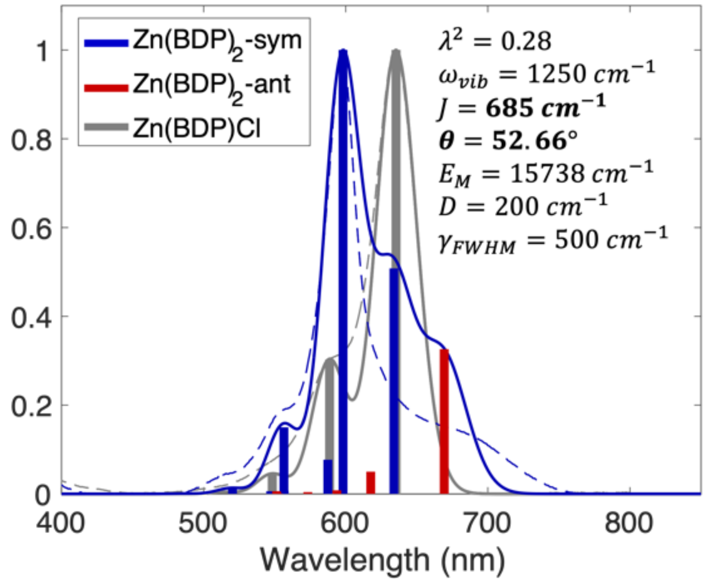

Figure S12. Absorption spectrum of $Zn(BDP)_2$ (solid, blue) simulated using the Frenkel-Holstein Hamiltonian (Eq. 2 - main text) and the parameters extracted from DFT/TDDFT calculations: r=5.97Å, θ=52.66° (J_{12}=685 cm^{-1}), |μ|=10.32 Debye, HR factor=0.28, ω_{vib}=1250 cm^{-1}, E_M=15,738 cm^{-1}, D=200 cm^{-1}. The optical dielectric constant was ε_{op}=2.23. The transitions (blue for e_{sym} and red for e_{ant} states) were broadened using Gaussians (FWHM=500 cm^{-1}). The experimental absorption spectra of Zn(BDP)Cl (grey) and $Zn(BDP)_2$ (blue) are shown with dashed lines.

Table S4. Twelve lowest energy excitonic states with their associated transition wavelengths and normalized absorbances, where the absorbance of 1.0 corresponds to the peak absorption (603.9 nm). The states were obtained using the parameters listed in the caption of Fig. S12. The transitions to the excitonic states corresponding to the normalized absorbance values greater than 0.1 are highlighted in grey and make the largest contribution to the linear spectrum.

	λ (nm)	Norm. abs
e_{ant1}	**676.5**	**0.326**
e_{sym1}	**640.4**	**0.507**
e_{ant}	623.8	0.049
e_{sym2}	**603.9**	**1.000**
e_{ant}	599.5	0.008
e_{sym}	592.9	0.077
e_{ant}	578.7	0.004
e_{sym}	561.7	0.007
e_{sym3}	**561.5**	**0.151**
e_{ant}	557.7	0.001
e_{ant}	556.3	0.006
e_{sym}	552.0	0.006

Following the initial simulation (Fig. S12, Table S4), the dihedral angle θ and distance r were adjusted using the *fminsearch* function in Matlab, while D was adjusted manually, in order to obtain the best fit to the experimental spectra. The values that gave the best fit were: θ=43.5°, r=5.62 Å (J_{12}=982 cm^{-1}), D=325 cm^{-1}. The 12 lowest energy states after the optimization are reported in Table S5, and the corresponding simulated spectrum is shown in Fig. 3 (main text).

Table S5. Twelve lowest energy excitonic states with the associated transition wavelengths and normalized absorbances, where the absorbance of 1.0 corresponds to the peak absorption (597.0 nm). The states were obtained using the following optimized parameters: r=5.62Å, θ=43.5° (J_{12}=982 cm^{-1}), HR factor=0.28, ω_{vib}=1250 cm^{-1}, $|\mu|$=10.32 Debye, E_M=15,738 cm^{-1}, and D=325 cm^{-1}. The optical dielectric constant was ε_{op}=2.23. The transitions to the excitonic states corresponding to the normalized absorbance values greater than 0.1 are highlighted in grey and are shown as solid lines in Fig. 3b of the main text.

	**λ (nm)**	**Norm. abs**
e_{ant1}	**688.4**	**0.161**
e_{sym1}	**644.9**	**0.176**
e_{ant}	633.9	0.025
e_{ant}	603.2	0.002
e_{sym2}	**597.0**	**1.000**
e_{sym}	596.8	0.027
e_{ant}	587.3	0.002
e_{sym}	565.0	0.006
e_{ant}	560.8	0.000
e_{sym3}	**555.5**	**0.150**
e_{sym}	555.3	0.002
e_{ant}	550.7	0.003

To demonstrate the need to include two-particle states in the simulations we also simulated the linear spectrum using only one particle states. We used the same optimization procedure described above and in the main text to determine the optimized values of r and θ to be r =5.70Å and θ=38.6° which gives a J_{12}=1012 cm^{-1}. The simulated spectrum using only one-particle states is shown in Fig. S13. Comparing Fig. S13 and Fig. 3a in the main text, it is clear that the inclusion of the two-particle states gives a much better agreement with the experimental spectrum.

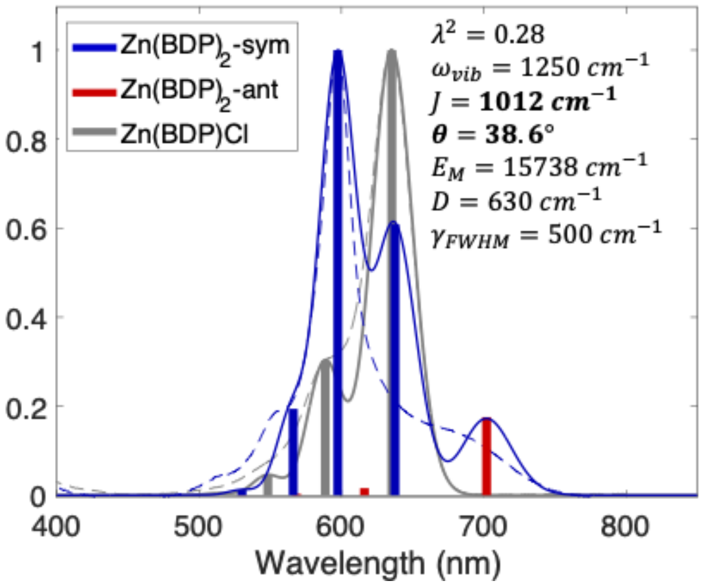

Figure S13. Absorption spectrum of $Zn(BDP)_2$ (solid, blue) simulated using the Frenkel-Holstein Hamiltonian using a one-particle basis set. The parameters used to simulate the spectra were: r= 5.70 Å, θ= 38.6°, (giving a J_{12}=1012 cm^{-1}), ε_{op}=2.23, |**μ**|=10.32 Debye, HR factor=0.28, ω_{vib}=1250 cm^{-1}, E_M=15,738 cm^{-1}, D=630 cm^{-1}. The transitions (blue for e_{sym} and red for e_{ant} states) were broadened using Gaussians (FWHM=500 cm^{-1}). The experimental absorption spectra of Zn(BDP)Cl (grey) and $Zn(BDP)_2$ (blue) are shown as dashed lines for comparison.

SI 5. Waiting Time (t_2) Dependent 2DES Spectra of Zn(BDP)Cl & $Zn(BDP)_2$

The normalized t_2-dependent 2DES spectra of Zn(BDP)Cl are shown in Fig. S14. The incoming laser pulses are tuned to overlap with the $S_{00} \rightarrow S_{10}$ transition and a small portion of the $S_{00} \rightarrow S_{11}$ transition (the Franck-Condon active mode at 1250 cm^{-1}), and these two transitions contribute to the main peak in the 2DES spectra. We note that other lower frequency Franck-Condon (FC) active modes could also contribute to the 2DES peak. At later waiting times, the 2DES spectral shape appears box-like due to the unresolved cross peaks between the $S_{00} \rightarrow S_{10}$ transition and the FC active modes. We also note that the different FC transitions contributing to the main peak oscillate as a function of t_2 waiting time, leading to λ_1,λ_3 dependent amplitude modulations of the main peak. This is consistent with previous 2DES studies on laser dyes and BODIPY chromophores.

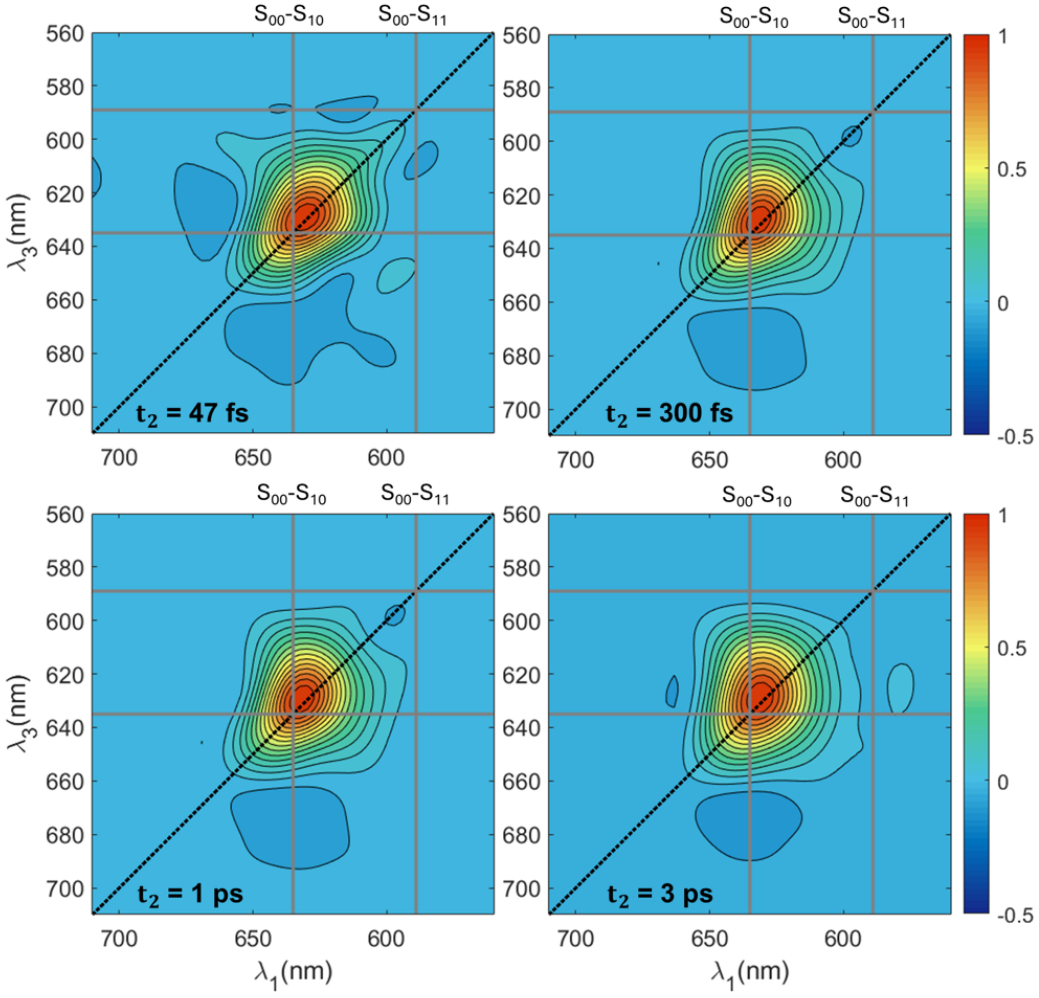

Figure S14. Normalized 2DES spectra of Zn(BDP)Cl in pyridine at representative waiting times of 47 fs, 300 fs, 1 ps, and 3 ps. The maxima of the S_{00}-S_{10} and S_{00}-S_{11} transitions from modeling the linear absorption spectrum are indicated as solid grey lines superposed on the 2DES spectra.

The t_2 dependent 2DES spectra of $Zn(BDP)_2$ are shown in Fig. S15. The 2DES spectra resolve cross peaks associated with the excitonically coupled states as discussed in the main text. Given the tuning of the incoming laser pulses (SI 1) the 2DES spectra only monitor the 580-700 nm region, so the 2DES spectra only excite and probe the GSB transitions at early times. In principle, projecting the 2DES spectrum onto the λ_3 axis would result in the corresponding pump-probe spectrum taken under the same experimental conditions (the same pulse tunings and energies). For our current experiments, we have used different pump and probe pulse tunings for the fsTA and the 2DES as we use the different techniques to characterize different aspects of the photophysics. Though a direct comparison was not made between the two experiments, we do find that the results are consistent between the fsTA and the 2DES, where the GSB does not significantly decay over the first 3 ps.

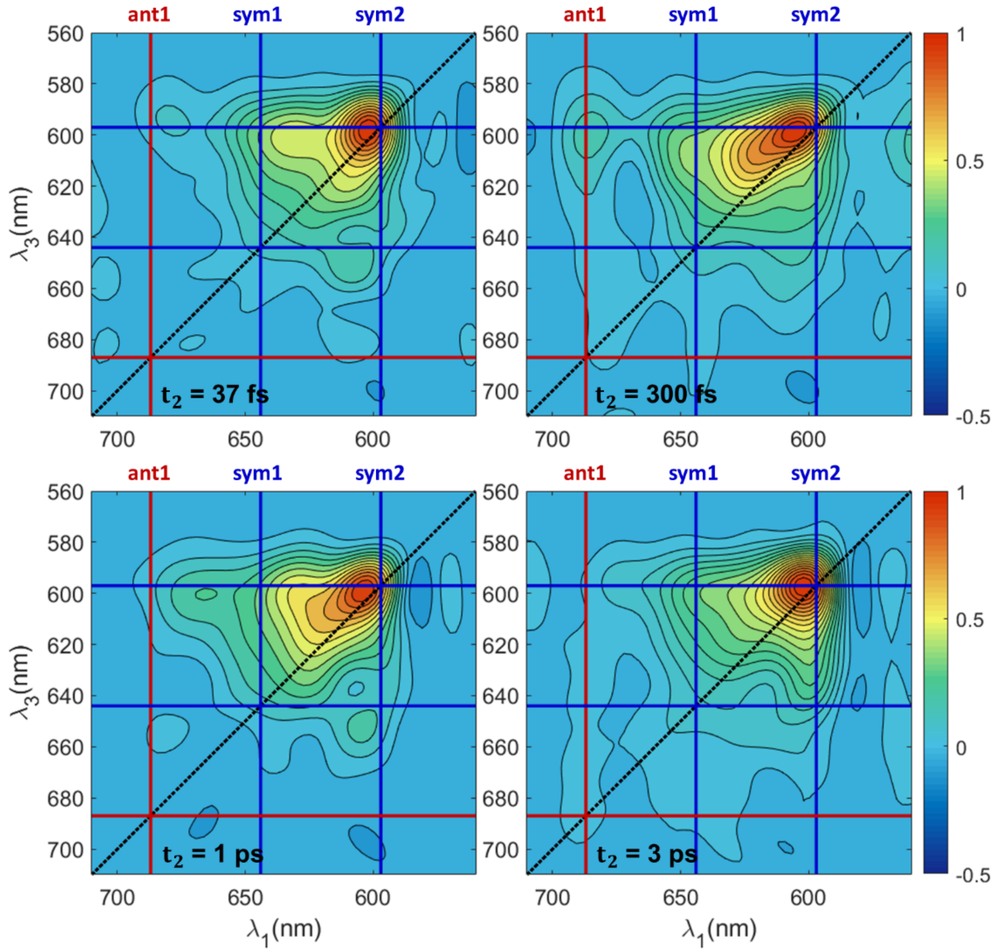

Figure S15. Normalized 2DES spectra of $Zn(BDP)_2$ in TolH at representative waiting times of 37 fs, 300 fs, 1 ps, and 3 ps. The transitions predicted by the Frenkel-Holstein model (Main text Fig. 3) are noted as solid red and blue lines superposed on the 2DES spectra.

SI 6. fsTA Spectra of $Zn(BDP)_2$ in Et_2O and cHex

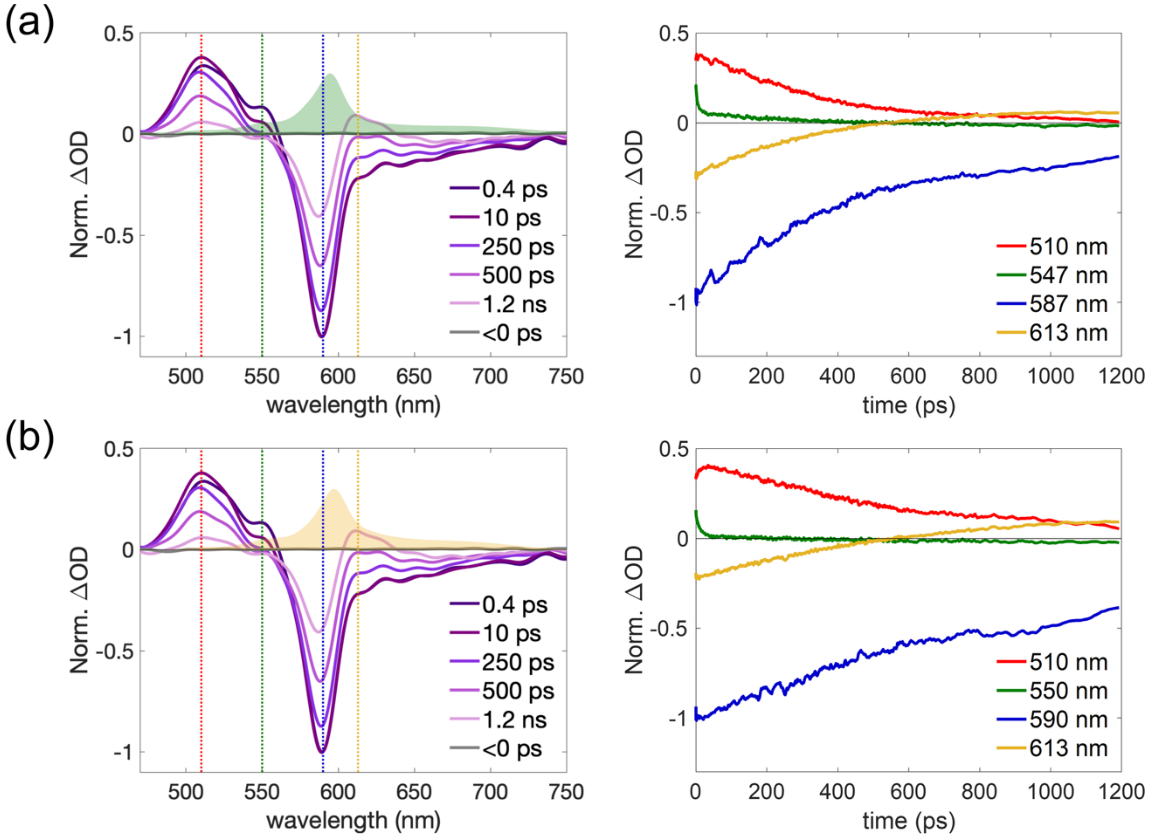

Figure S16. fsTA spectra and temporal ΔOD traces at selected wavelengths of $Zn(BDP)_2$ in (a) Et_2O and (b) cHex. The linear absorption spectra are illustrated as shaded areas.

SI 7. Decay Associated Spectra (DAS) and Evolution Associated Spectra (EAS)

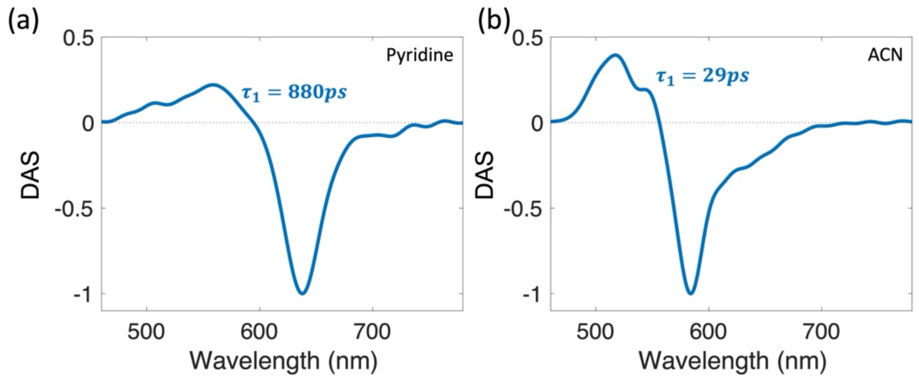

Figure S17. DAS extracted from fsTA of (a) Zn(BDP)Cl in pyridine and (b) $Zn(BDP)_2$ in acetonitrile (MeCN)

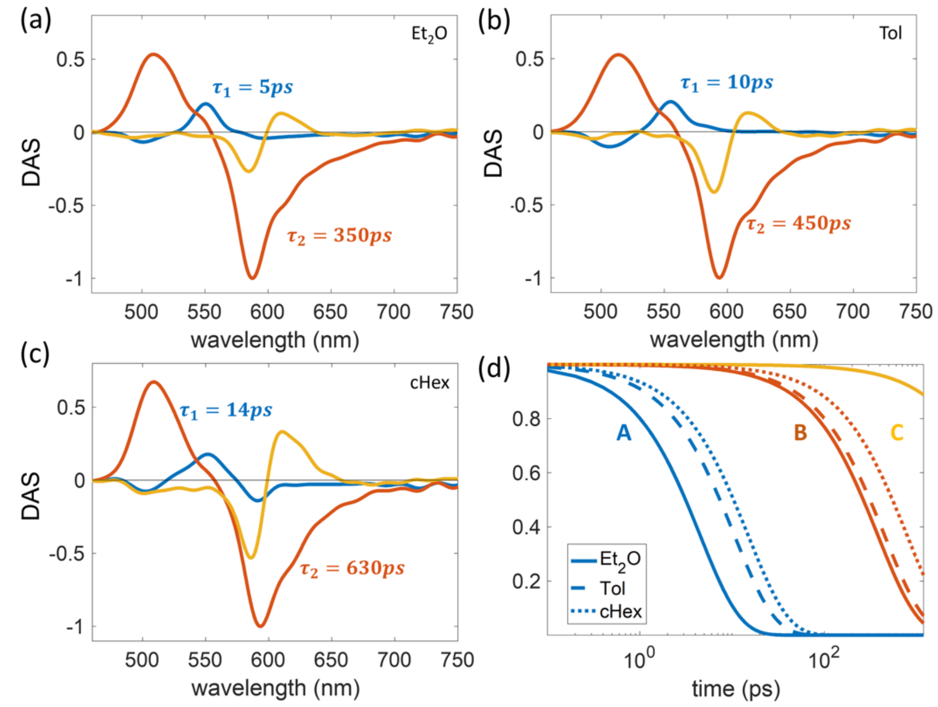

Figure S18. (a-c) Decay associated spectra (DAS) extracted from the fsTA of $Zn(BDP)_2$ in diethyl ether (Et_2O), toluene (TolH) and cyclohexane (cHex). (d) The evolution of each DAS component shows the lifetime trend is associated with the polarity of the solvents.

SI 8. Electrochemical Data

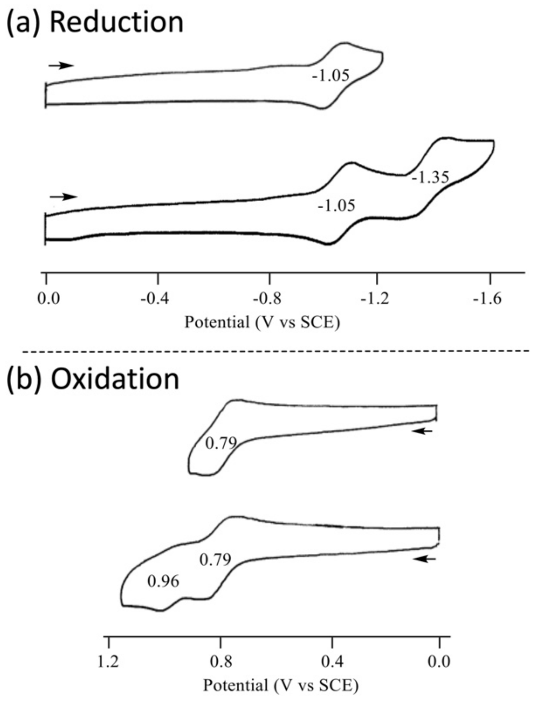

Figure S19. Cyclic voltammograms of $Zn(BDP)_2$ in dichloromethane (DCM) containing 0.1 M Tetra-n-butylammonium perchlorate (TBAP). The reductions (a) and oxidations (b) are electrochemically reversible. The potentials are: $E_{1/2}$ = -1.05 and -1.35 V *vs* SCE for reduction and at $E_{1/2}$ = 0.79 and 0.96 V *vs* SCE for oxidation.

SI 9. nsTA Spectra of $Zn(BDP)_2$

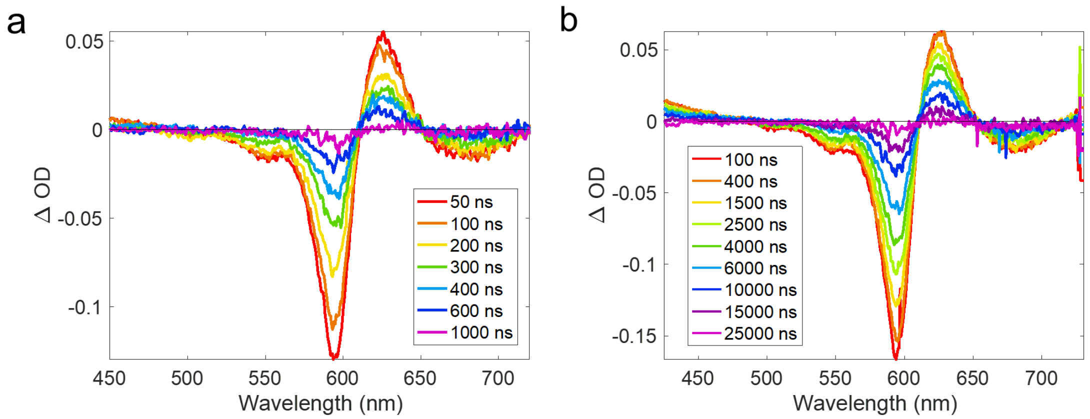

Figure S20. nsTA spectra of $Zn(BDP)_2$ in (a) aerated and (b) deoxygenated TolH at 23°C. λ_{ex} = 532 nm.

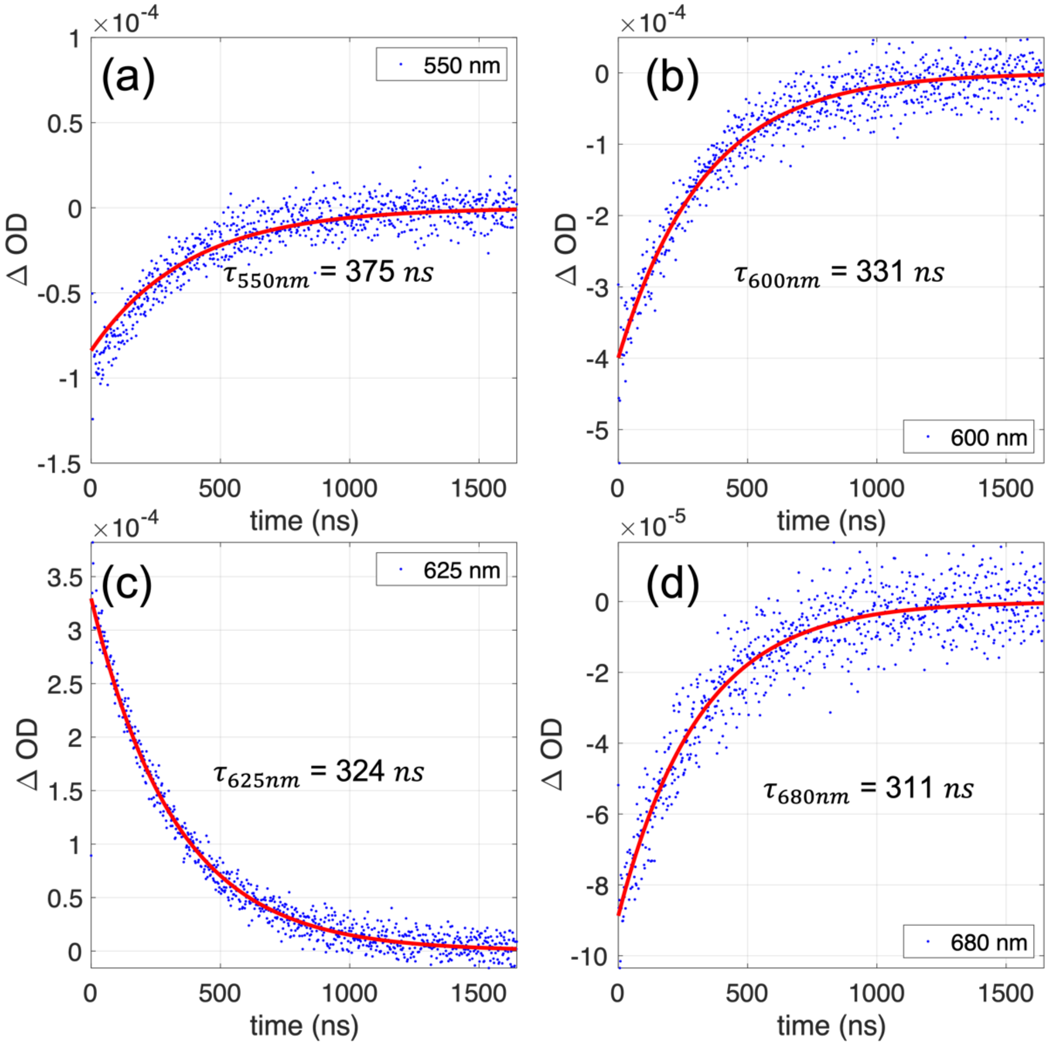

Figure S21. nsTA traces of $Zn(BDP)_2$ in in air-saturated toluene monitored at (a) 550 nm, (b) 600 nm, (c) 625 nm, and (d) 680 nm measured using a single wavelength nsTA spectrometer with 532 nm excitation (Magnitude instruments, enVISion). The data were fit to a single exponential decay model (red line).

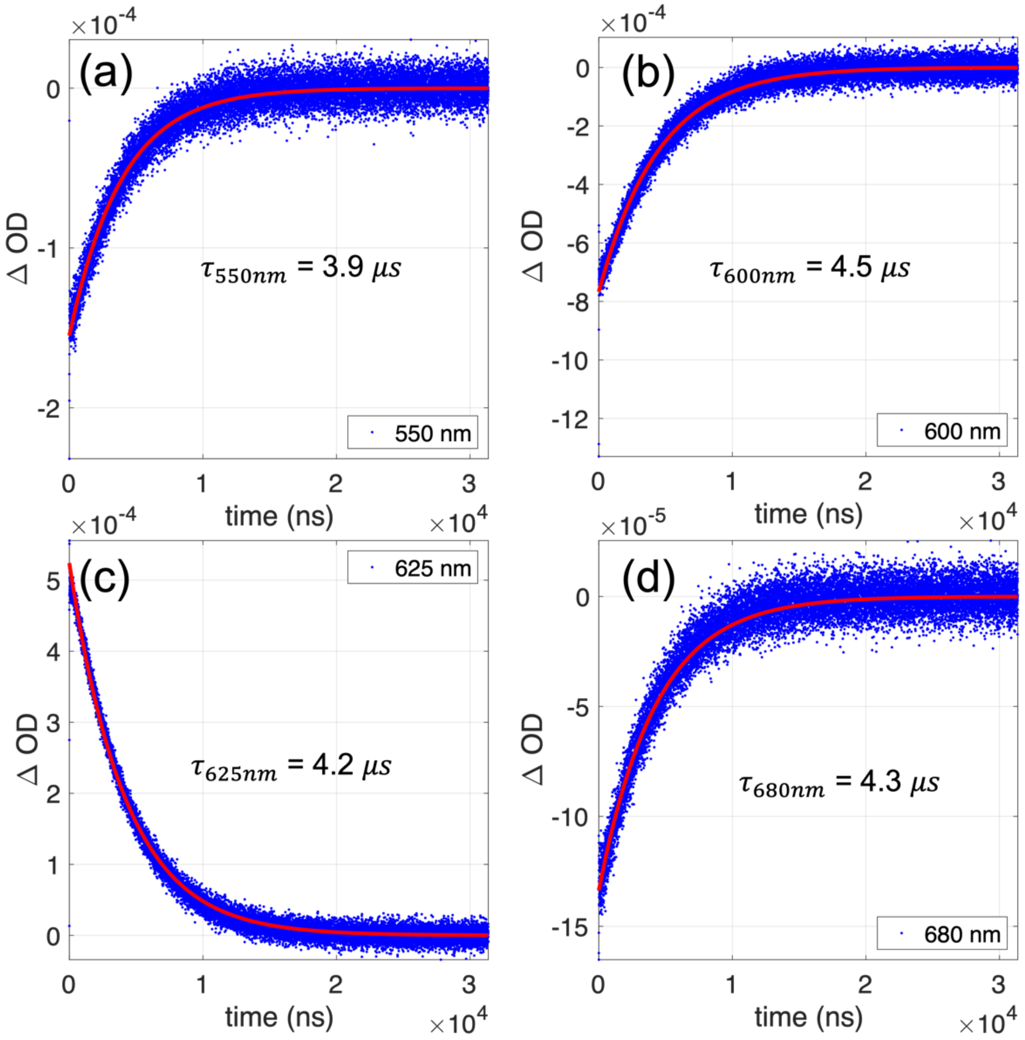

Figure S22. nsTA traces of $Zn(BDP)_2$ in N_2-purged toluene at (a) 550 nm, (b) 600 nm , (c) 625 nm, and (d) 680 nm measured using a single wavelength nsTA spectrometer with 532 nm excitation (Magnitude instruments, enVISion). The data were fit with a single-exponential decay function (red line).

Table S6. Lifetimes of the nsTA decays of Zn(BDP) in toluene (λ_{ex}=532 nm).

	550 nm	600 nm	625 nm	680 nm	Mean (STD)
Air (ns)	375	331	324	311	335 (28)
N_2 (μs)	3.9	4.5	4.2	4.3	4.2 (0.3)

SI 10. Kinetic Simulations

Kinetic simulations were performed using a custom-written code (C/C++, CodeBlocks). The system was modeled as described in the kinetic scheme shown in Fig. 7 (main text; also shown in the inset). The resulting lifetimes and rate constants (s^{-1}) are shown in the tables below.

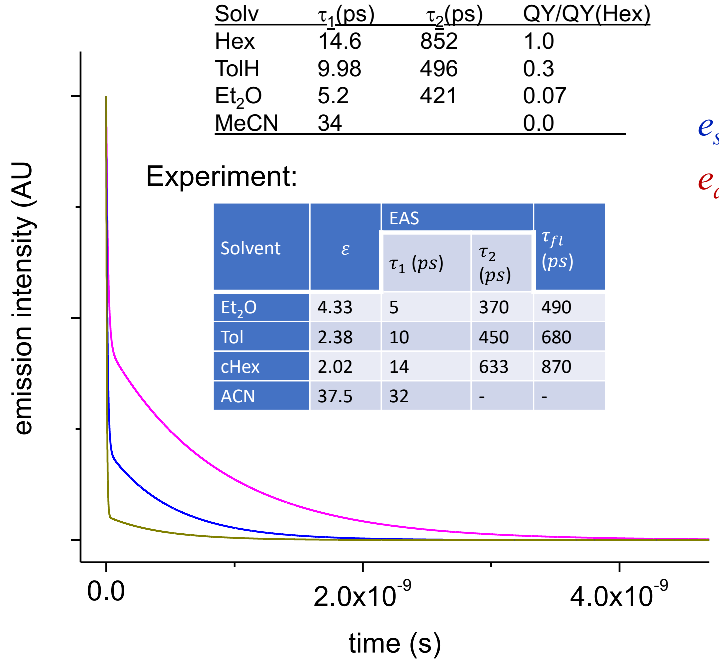

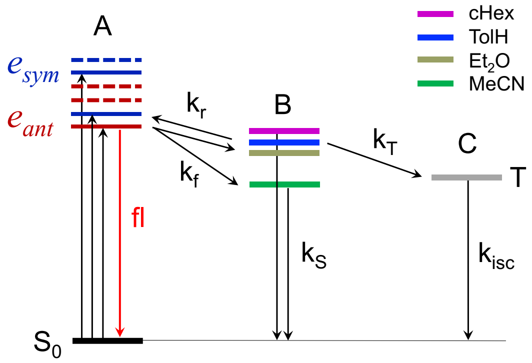

$k_{fl} = 10^8\ s^{-1}$

$k_{isc} = 10^6\ s^{-1}$

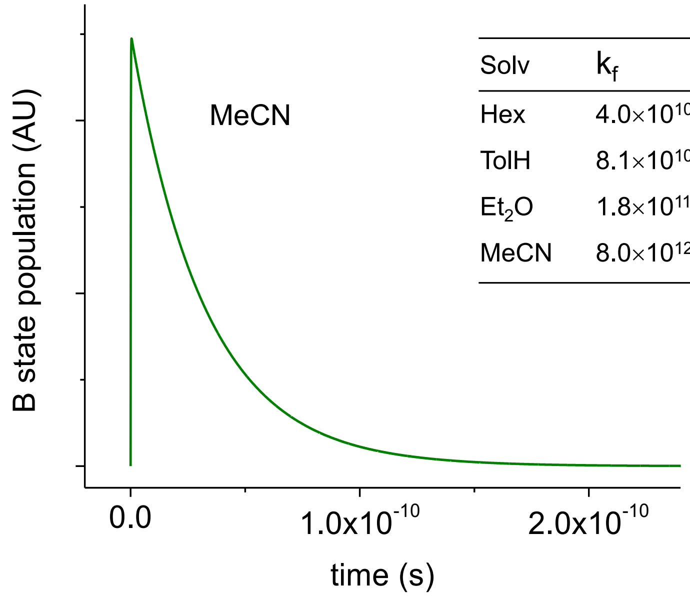

Solv	k_f	k_r	k_S	k_T
Hex	4.0×10^{10}	3.0×10^{10}	1.0×10^{9}	1.0×10^{9}
TolH	8.1×10^{10}	2.0×10^{10}	1.4×10^{9}	1.0×10^{9}
Et_2O	1.8×10^{11}	1.0×10^{10}	1.5×10^{9}	1.0×10^{9}
MeCN	8.0×10^{12}	1.0×10^{9}	3.0×10^{10}	1.0×10^{9}

$k_{fl} = 10^8\ s^{-1}$

$k_{isc} = 10^6\ s^{-1}$

SI 11. Time-Resolved Fluorescence Measurements of $Zn(BDP)_2$

<u>Cyclohexene (cHex)</u>

in cyclohexane ex=525nm emi = 720-800nm

Model: Exp. [Reconv.] (Exponential)
Plotted Data Set #0 Decay: "\..\cHex.sdt" (0)
Plotted Data Set #0 IRF: "\..\IRF-H7422-750mV.sdt" (0)
X^2(reduced): 1.1172 ; Fitted Data Points: 574

Main Plot

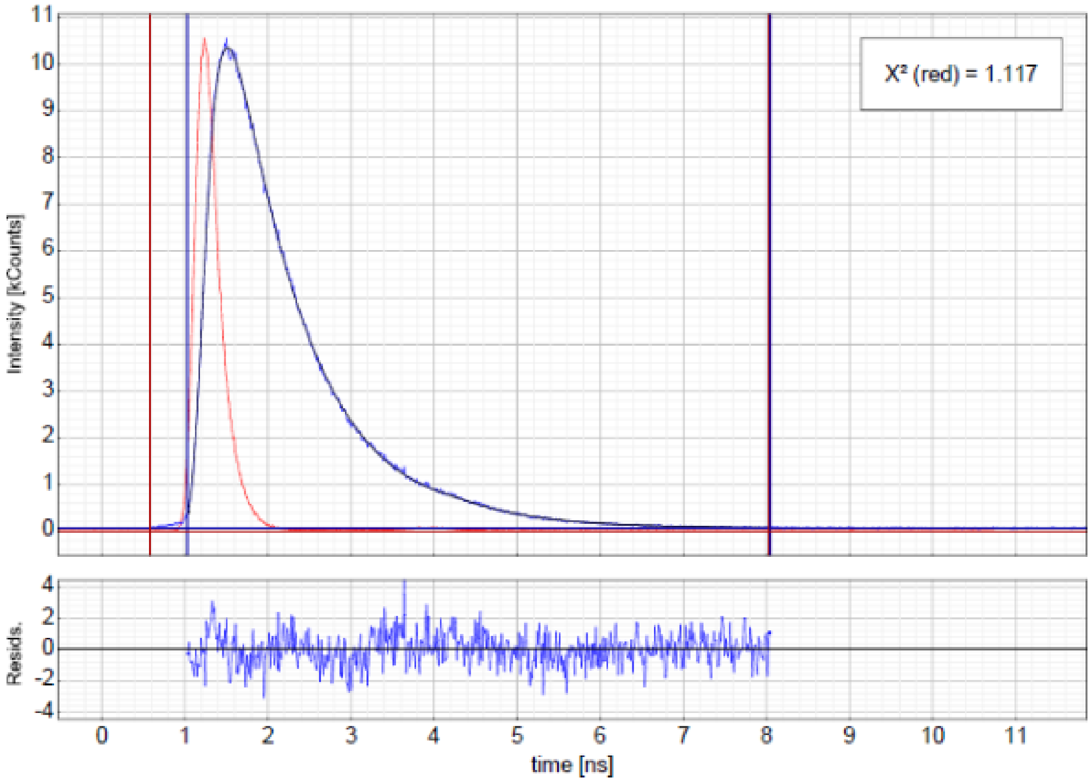

$$I(t) = \int_{-\infty}^{t} IRF(t') \sum_{i=1}^{n} A_i \, e^{-\frac{t-t'}{\tau_i}} \, dt'$$

Parameter	Value	Conf. $_{Lower}$	Conf. $_{Upper}$	Conf. $_{Estimation}$
A_1 [Cnts]	16175.8	-61.5	+61.5	Fitting
wi [ns]	0.87010	-0.00258	+0.00258	Fitting
Bkgr. $_{Dec}$ [Cnts]	62.00	-2.67	+2.67	Fitting
Bkgr. $_{IRF}$ [Cnts]	4.5	---	---	<none>
Shift $_{IRF}$ [ns]	-1.10574	-0.00148	+0.00148	Fitting
Period $_{Rep}$ [ns]	12.500	---	---	<none>

<u>Diethyl ether</u>

in ether ex=525nm emi = 720-800nm

Model: Exp. [Reconv.] (Exponential)
Plotted Data Set #0 Decay: "\..\ether.sdt" (0)
Plotted Data Set #0 IRF: "\..\IRF-H7422-750mV.sdt" (0)
X^2(reduced): 2.4871 ; Fitted Data Points: 402

Main Plot

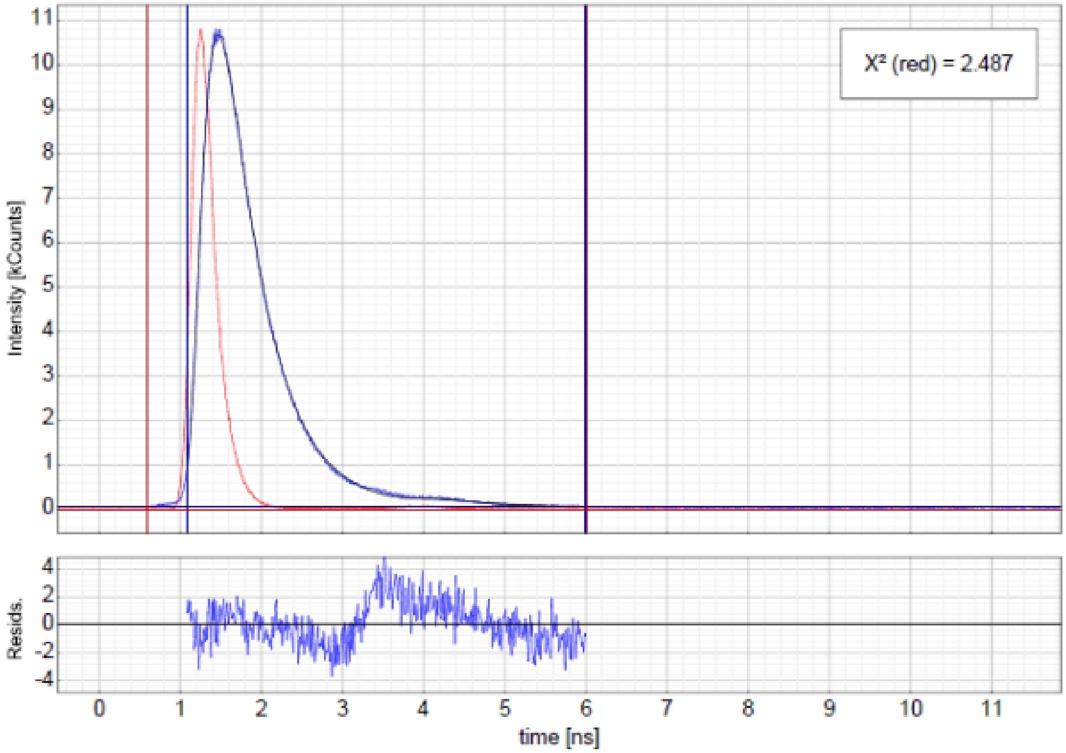

$$I(t) = \int_{-\infty}^{t} IRF(t') \sum_{i=1}^{n} A_i \, e^{-\frac{t-t'}{\tau_i}} \, dt'$$

Parameter	Value	Conf. Lower	Conf. Upper	Conf. Estimation
A_1 [Cnts]	20214	-115	+115	Fitting
τ_1 [ns]	0.48990	-0.00216	+0.00216	Fitting
Bkgr. Dec [Cnts]	56.98	-3.76	+3.76	Fitting
Bkgr. IRF [Cnts]	4.5	---	---	<none>
Shift IRF [ns]	-1.08839	-0.00176	+0.00176	Fitting
Period Rep [ns]	12.500	---	---	<none>

<u>Toluene</u>

in Tolune second trial   ex=525nm   emi = 720-800nm

Model: Exp. [Reconv.] (Exponential)
Plotted Data Set #0 Decay: "\..\TolH2.sdt" (0)
Plotted Data Set #0 IRF: "\..\IRF-H7422-750mV.sdt" (0)
X^2(reduced): 7.3041 ; Fitted Data Points: 413

Main Plot

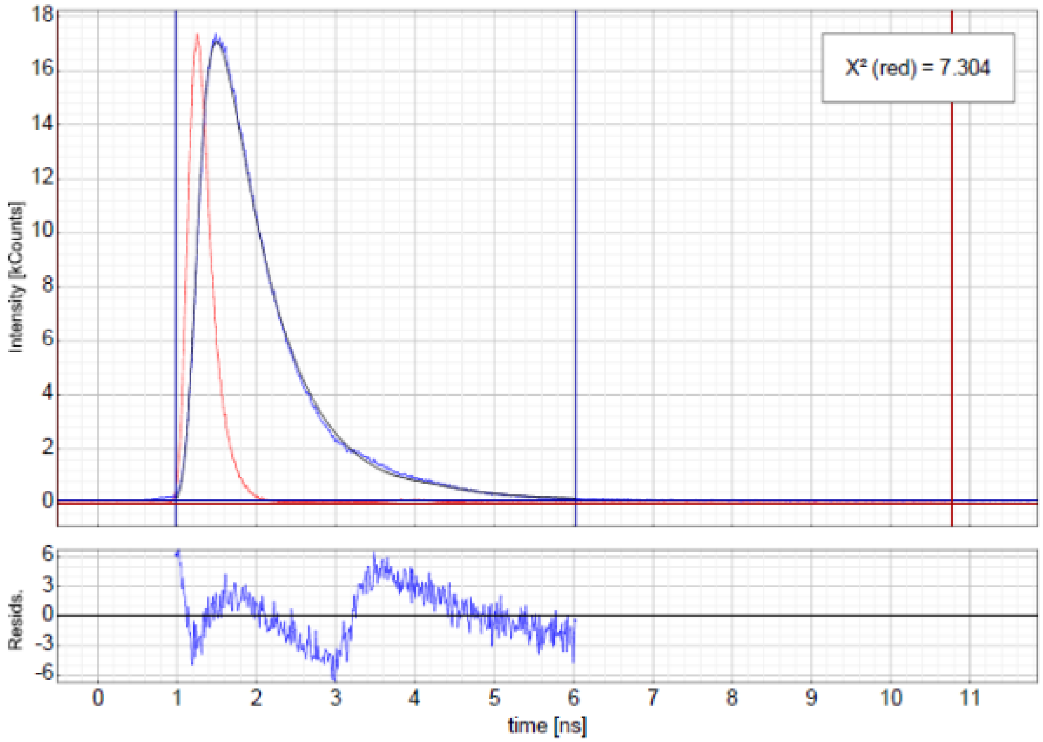

$$I(t) = \int_{-\infty}^{t} IRF(t') \sum_{i=1}^{n} A_i \, e^{-\frac{t-t'}{\tau_i}} \, dt'$$

Parameter	Value	Conf. $_{Lower}$	Conf. $_{Upper}$	Conf. $_{Estimation}$
A_1 [Cnts]	28705	-201	+201	Fitting
w_1 [ns]	0.67832	-0.00375	+0.00375	Fitting
Bkgr. $_{Dec}$ [Cnts]	114.5	-10.8	+10.8	Fitting
Bkgr. $_{IRF}$ [Cnts]	4.2	---	---	<none>
Shift $_{IRF}$ [ns]	-1.08895	-0.00235	+0.00235	Fitting
Period $_{Rep}$ [ns]	12.500	---	---	<none>
